# The Epstein-Barr virus deubiquitinase BPLF1 regulates stress-induced ribosome UFMylation and reticulophagy

**DOI:** 10.1080/15548627.2024.2440846

**Published:** 2025-01-22

**Authors:** Jiangnan Liu, Noemi Nagy, Carlos Ayala-Torres, Solenne Bleuse, Francisco Aguilar-Alonso, Ola Larsson, Maria G. Masucci

**Affiliations:** a Department of Cell and Molecular Biology, Karolinska Institutet, Stockholm, Sweden; b Science for Life Laboratory, Department of Oncology-Pathology, Karolinska Institutet, Stockholm, Sweden

**Keywords:** Macroautophagy, UFM1, viral DUB, Ribosome, EBV

## Abstract

The synthesis of membrane and secreted proteins is safeguarded by an endoplasmic reticulum-associated ribosome quality control (ER-RQC) that promotes the disposal of defective translation products by the proteasome or via a lysosome-dependent pathway involving the degradation of portions of the ER by macroautophagy (reticulophagy). The UFMylation of RPL26 on ER-stalled ribosomes is essential for activating the ER-RQC and reticulophagy. Here, we report that the viral deubiquitinase (vDUB) encoded in the N-terminal domain of the Epstein-Barr virus (EBV) large tegument protein BPLF1 hinders the UFMylation of RPL26 on ribosomes that stall at the ER, promotes the stabilization of ER-RQC substrates, and inhibits reticulophagy. The vDUB did not act as a de-UFMylase or interfere with the UFMylation of the ER membrane protein CYB5R3 by the UFL1 ligase. Instead, it copurified with ribosomes in sucrose gradients and abrogated a ZNF598- and LTN1-independent ubiquitination event required for RPL26 UFMylation. Physiological levels of BPLF1 impaired the UFMylation of RPL26 in productively EBV-infected cells, pointing to an important role of the enzyme in regulating the translation quality control that allows the efficient synthesis of viral proteins and the production of infectious virus.

**Abbreviation**: BPLF1, BamH1 P fragment left open readingframe-1; CDK5RAP3, CDK5regulatory subunit associated protein 3; ChFP, mCherry fluorescent protein; DDRGK1, DDRGKdomain containing 1; EBV, Epstein-Barr virus; eGFP, enhancedGFP; ER-RQC, endoplasmicreticulum-associated ribosome quality control; LCL, EBV-carryinglymphoblastoid cell line; GFP, green fluorescent protein; RQC, ribosome quality control; SRP, signal recognition particle; UFM1, ubiquitin fold modifier 1; UFL1, UFM1 specific ligase 1.

## Introduction

The integrity of the cellular proteome is preserved by a proteostasis network that, through multiple factors and cellular machinery, directs the production, folding, trafficking, and degradation of proteins [1]. Because protein biosynthesis is highly complex and error-prone, eukaryotes have evolved protein quality-control checkpoints that trigger the repair or elimination of faulty products. Defective translation products generated by the stalling of cytosolic ribosomes at specific mRNA sequences are eliminated via a process known as ribosomal quality control (RQC) that involves the recognition of collided ribosomes and ubiquitination of the 40S proteins RPS10/eS10 and RPS20/uS10 by ZNF598 (zink finger protein 598, E3 ubiquitin ligase), which promotes ribosome disassembly and the subsequent ubiquitination of the 60S-associated nascent polypeptide by LTN1 (listerin E3 ubiquitin protein ligase 1), followed by degradation by the proteasome [2–7]. The stalling of ER-associated ribosomes that translate secretory or membrane proteins causes clogging of the SEC61 translocon and activates an ER-associated ribosome quality control (ER-RQC, also known as translocon-associated quality control, TAQC) [8–11] that promotes the degradation of the aberrant polypeptides by the proteasome [8,12] or by the lysosome [11,13,14]. Aberrant translation products that accumulate in the ER due to failure of this quality control trigger the unfolded protein response/UPR that restrains protein production, activates the protein folding capacity of the ER, and triages terminally misfolded proteins for disruption by the proteasome via ER-associated degradation/ERAD [15,16], or by the lysosome [17,18].

Activation of the ER-RQC was shown to trigger the destruction of portions of the ER by macroautophagy (hereafter referred to as reticulophagy), which involves the physical separation and sequestration of segments of the ER within double-membrane autophagosomes that fuse with lysosomes for content recycling [13,19–21]. The process relies on specific reticulophagy receptors that are either cytosolic or ER-membrane proteins characterized by the presence of LC3-interacting regions/LIRs in their cytosolic domains [19,22]. Recent studies have revealed the involvement of the ubiquitin-like protein UFM1 (ubiquitin fold modifier 1) in the regulation of ER-RQC and reticulophagy [23–28]. Like ubiquitin, UFM1 is covalently attached to target proteins via an enzymatic cascade involving the activating enzyme UBA5 (ubiquitin like modifier activating enzyme 5; E1), the conjugating enzyme UFC1 (ubiquitin-fold modifier conjugating enzyme 1; E2), and the ligase UFL1 (UFM1 specific ligase 1; E3) that mediates the transfer of UFM1 from the E2 to the substrate [29]. The UFMylation machinery is recruited to the ER by the adaptor protein DDRGK1/UFBP1 (DDRGK domain containing 1) [23–28]. The interaction with DDRGK1 activates the E3 ligase [30,31]. In addition, the activity of the ligase is regulated by binding to CDK5RAP3/LZAP/C53 (CDK5 regulatory subunit associated protein 3) [30,31], which also serves as a specific receptor for ribosome stress-induced reticulophagy [27]. The ER-associated ligase UFMylates the 60S subunit protein RPL26/uL24 [24–26] on ribosomes that stall at the ER [12], and several ER-localized proteins, including the oligosaccharyltransferase complex subunit RPN1 [30,31], and CYB5R3 (cytochrome b5 reductase 3) that was recently proposed as a specific regulator of reticulophagy [28]. The molecular triggers of UFMylation and the mechanism by which UFMylation leads to activation of the ER-RQC and reticulophagy are poorly understood. It was recently proposed that the UFMylation of RPL26 May be required to relax the ribosome-translocon junction to allow the VCP/p97-dependent extraction of the translocon-associated aberrant polypeptide to the cytosol and subsequent degradation by the cytosolic RQC [12]. In addition, UFMylation of the ribosome or other ER-associated proteins may serve as a marker for the recruitment of proteins involved in autophagosome biogenesis, as suggested by the finding that CDK5RAP3 binds to both UFM1 and LC3 via a noncanonical LC3-interacting region motif [12,22,27]. Loss of the UFMylation machinery [32], DDRGK1 [33], or CDK5RAP3 [27] was shown to trigger ER stress in mammalian cells, which highlights a tight connection between UFMylation, ER-RQC, reticulophagy, and the maintenance of ER homeostasis.

During viral infection, the incoming viruses hijack the cellular translation machinery to produce large amounts of viral proteins that overload the ER with polypeptides that are often large, multidomain, and difficult to fold, which may cause ER stress [34]. Furthermore, many RNA viruses remodel the ER membranes to create microenvironments permissive to virus replication [35], highlighting the need to evade the control mechanisms that maintain the functionality and integrity of this cellular organelle. The strategies adopted by viruses to remodel the function of the ER are poorly understood, but accumulating evidence points to significant differences depending on the type of virus and mode of virus replication [36–38]. Several viral proteins, including the Epstein-Barr virus (EBV)-encoded LMP1 [39] and BMLF1 [40], were shown to interact with components of the unfolded protein response and ER-associated degradation machinery to inhibit their intrinsic antiviral properties or repurpose the activity toward virus production [41]. Much less is known about viral interference with the ER-RQC and reticulophagy.

EBV is a human lymphotropic herpesvirus that causes clinically silent primary infections in childhood, followed by the establishment of a lifelong carrier state associated with diverse pathologies. Best known is the involvement of EBV in the pathogenesis of lymphoid and epithelial cell malignancies [42], while impaired control of the productive virus cycle has been reported before the onset of EBV-associated malignancies and in patients suffering from autoimmune diseases, such as multiple sclerosis, rheumatoid arthritis, and lupus erythematosus [43]. During productive EBV infection, the cellular mRNA translation machinery is reprogrammed to favor the production of viral proteins required for the building of new virus. We have previously shown that the ubiquitin deconjugase (vDUB) encoded in the N-terminal domain of the large tegument protein BPLF1 participates in the translational reprogramming by counteracting the activity of the RQC ligases ZNF598 and LTN1, and promoting the activation of EIF2AK4/GCN2 (eukaryotic translation initiation factor 2 alpha kinase 4)-dependent integrated stress responses, which together enhance the translation of viral mRNAs [44]. Here, we report on the capacity of the vDUB to counteract the UFMylation of RPL26 upon ribosomal stress. Inhibition of this branch of the RQC correlated with the rescue of a model ER-RQC substrate from both proteasome- and lysosome-dependent degradation and inhibition of reticulophagy. We found that the physiologically expressed active enzyme inhibits the UFMylation of stalled ribosomes in a productively infected EBV-carrying lymphoblastoid cell line (LCL), pointing to a broad contribution of the vDUB in restricting the antiviral effects of ribotoxic stress responses.

## Results

### BPLF1 participates in protein complexes involved in mRNA translation and the ER-associated protein quality control

**Figure 1.**
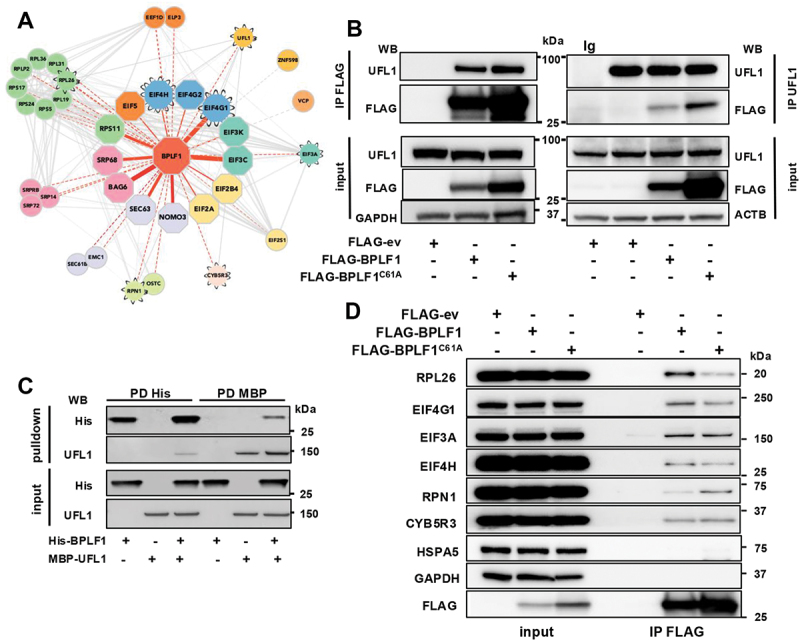
BPLF1 interacts with the UFM1 ligase UFL1 and putative UFL1 substrates. (A) curated STRING network diagram of the BPLF1 interacting proteins identified by co-immunoprecipitation and mass spectrometry. The functional annotation of the interacting proteins is color-coded: green, ribosome subunits; yellow, subunits of the EIF2 translation pre-initiation complex; turquoise, subunits of the EIF3 translation pre-initiation complex; blue, translation initiation complex; orange, translation elongation factors; pink, signal recognition particle (SRP) and SRP receptor (SRPRB) involved in the recognition and targeting of signal-sequence-tagged proteins the ER; lilac, ER translocon complex that mediates forward and retrograde transport across the ER; lime, translocon-associated N-oligosaccharyltransferase (OST) complex that links high mannose sugars to the asn-X-Ser/Thr consensus motif of ER-translocated polypeptides; light pink, ER protein CYB5R3 involved in the regulation of reticulophagy, light orange, UFM1 and ZNF598 ligases. Waved borders indicate putative UFMylation substrates. The thickness of the red connecting lanes indicates Log_2_ fold change: 4 points ≥ 10: 3 points ≥ 7–10; 2 points ≥ 5–7; 1 point ≥ 3–5; dotted lines ≥ 1.5–3. (B) reciprocal co-immunoprecipitation assays illustrate the interaction of BPLF1 with the UFL1 ligase. Lysates of HEK293T cells transfected with plasmids expressing FLAG-ev, BPLF1 or BPLF1^C61A^ were immunoprecipitated with either anti-FLAG coated beads or antibodies to UFL1 followed by capture with GammaBind^TM^ plus Sepharose^TM^ (cytiva 17,088,601). An isotype-matched immunoglobulin control (ig) was included in the UFL1 immunoprecipitation to verify specificity. Immunoblots were probed with the indicated antibodies. Blots from one representative experiment out of three are shown in the figure. (C) affinity isolation assay illustrating the interaction of BPLF1 with the UFL1 ligase. Equimolar concentrations of purified bacterially expressed His-BPLF1 and MBP-UFL1 were mixed, and affinity isolations of the HIS and MBP tags were performed from equal aliquots. Western blots from one out of two independent experiments are shown in the figure. The involvement of contaminating nucleic acids or nonspecific interaction mediated by the MBP tag was not formally excluded. (D) Representative western bots that illustrate the interaction of BPLF1 with the putative UFMylation substrates identified in (A). FLAG immunoprecipitation was performed from extracts of HEK293T cells transfected with FLAG-ev, -BPLF1 or -BPLF1^C61A^ and immunoblots were probed with the indicated antibodies. Each interaction was validated in at least two independent co-immunoprecipitation experiments.

We have previously shown that the vDUB encoded in the N terminus (aa 1–235) of the EBV large tegument protein BPLF1 is released by CASP1 (caspase 1) cleavage during productive infection and the active enzyme localizes in the nucleus and cytoplasm of the infected cells [45]. Using an immunoprecipitation and mass spectrometry approach, we found that the transfected vDUB domain interacts with a broad range of cellular proteins and protein complexes, pointing to a pleiotropic impact on cellular functions [46]. Analysis of the GO classes populated by proteins that interact with active and inactive versions of the vDUB where the catalytic Cys residue was mutated to Ala (hereafter BPLF1 and BPLF1^C61A^) revealed that a large proportion of the interacting proteins (169 out of 375 high-confidence interactors) are involved in RNA metabolism, ribosome biogenesis, mRNA translation, and ribotoxic stress responses [44]. To gain further insight into possible substrates and interacting partners, the mass spectrometry data were reanalyzed using less stringent exclusion parameters (see Materials and Methods), which confirmed the high prevalence of proteins involved in translation-related processes (540 out of 1190 proteins exhibiting Log_2_ fold change ≥ 1.5). STRING network analysis of the extended interactome identified a major hub of stronger (Log_2_ fold change between 3 to > 20, Figure 1A solid red lines) and weaker interactors (Log_2_ fold change between 1.5 to 3, Figure 1A dotted red lines) involved in mRNA translation, ER trafficking, and the ER stress response (for functional annotation see Table S1). The network included components of the 40S and 60S ribosome particles and several members of the translation pre-initiation, initiation, and elongation complexes [47], the SRP68, SRP72, and SRP14 subunits of the signal recognition particle (SRP) and SRPRB (SRP receptor subunit beta) that target nascent polypeptides to the ER [48], and the chaperone BAG6 (BAG cochaperone 6) that delivers tail-anchored proteins to the ER membrane [49]. Several ER membrane proteins were also found in the extended interactome, including the SEC63 and SEC61B subunit of the SEC61 translocon [50], the NOMO3 subunit of the multi-pass translocon/MTP complex [51], the EMC1 subunit of the endoplasmic reticulum membrane protein complex/EMC [51] that mediates transport across the ER and the biogenesis of multi-pass membrane proteins, the RPN1 (ribophorin I) and OSTC subunits of the translocon-associated N-oligosaccharyltransferase (OST) complex [52], and the cytochrome reductase CYB5R3 that participates in cholesterol biosynthesis and was recently proposed to regulate reticulophagy [28,51]. The interaction network also included the UFM1 ligase UFL1, which regulates the ER-RQC and reticulophagy responses [23–28], and the ubiquitin ligase ZNF598, which initiates the RQC upon the sensing of collided ribosomes [28,53].

The presence in the interaction network of components of the ribosome-translocon junction, UFL1, and several putative UFMylation substrates, including RPL26 [24], RPN1 [25], EIF3A, EIF2A, EIF4G1 [54], and CYB5R3 [28] (highlighted by waved borders in Figure 1A), suggests that BPLF1 May be involved in UFMylation-regulated processes at the ER. To address this possibility, we first sought to validate the interactions by co-immunoprecipitation using HEK293T cells transfected with FLAG-BPLF1, the catalytic mutant FLAG-BPLF1^C61A^, or the FLAG-empty vector (FLAG-ev) as transfection control. UFL1 was detected in the BPLF1 immunoprecipitates, independent of catalytic activity. Conversely, BPLF1 and BPLF1^C61A^ were found in the UFL1 immunoprecipitates (Figure 1B), confirming that the vDUB is associated with protein complexes containing the ligase. A weak interaction was also detected in reciprocal affinity isolation assays performed with bacterially expressed His-tagged BPLF1 and MBP-tagged UFL1, suggesting that the interaction may be direct (Figure 1C). Probing the FLAG immunoprecipitates with antibodies specific to the putative UFMylation substrates identified in Figure 1A confirmed their presence in BPLF1-containing complexes (Figure 1D), further corroborating the possible involvement of the vDUB in UFMylation-dependent cellular functions.

### BPLF1 counteracts the UFMylation of RPL26

**Figure 2.**
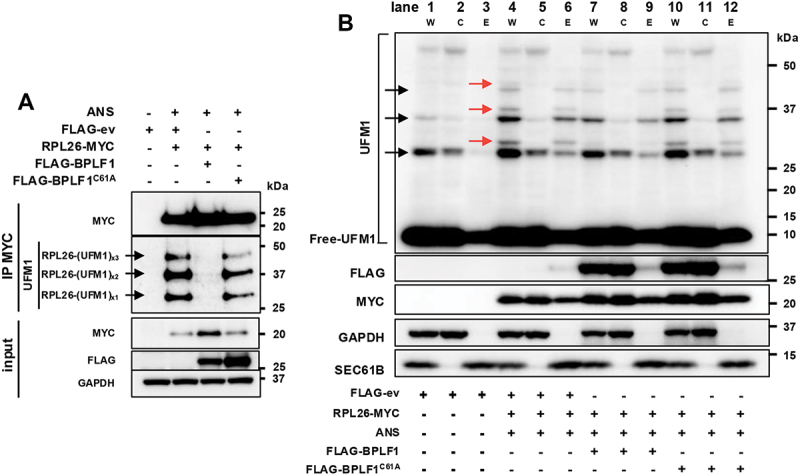
BPLF1 inhibits the UFMylation of RPL26 on ER-associated ribosomes. (A) BPLF1 inhibits the UFMylation of RPL26 in ANS-treated cells. Control HeLa cells and cells transfected with RPL26-MYC together with FLAG-ev, -BPLF1 or -BPLF1^C61A^ were treated with 50 ng/ml ANS for 1 h, followed by lysis under denaturing conditions to destroy non-covalent interactions and immunoprecipitation with anti-MYC coupled beads. Immunoblots from one representative experiment out of four are shown in the figure. (B) BPLF1 inhibits the ANS-induced UFMylation of RPL26 on ER-associated ribosomes. Total cell lysates (W), cytosolic (C), and ER membrane fractions (E) of ANS-treated cells cotransfected with RPL26-MYC and FLAG-ev, -BPLF1 or -BPLF1^C61A^ were probed with the indicated antibodies. Untreated FLAG-ev transfected cells were included to assess the abundance and subcellular localization of endogenous UFM1 adducts. UFMylated species of RPL26-MYC and endogenous RPL26 are indicated by red and black arrows, respectively. Immunoblots from one representative experiment out of three are shown in the figure.

RPL26 has been identified as the primary target of UFMylation on ribosomes that stall at the ER [24–26,55]. To investigate whether the vDUB regulates RPL26 UFMylation, HeLa cells were cotransfected with MYC-tagged RPL26 and FLAG-ev, -BPLF1 or -BPLF1^C61A^ for 24 h and then treated with the translation elongation inhibitor anisomycin (ANS) under conditions previously shown to induce random ribosome collision and activation of the RQC [53]. Preliminary experiments confirmed that treatment for 20 min with 50 ng/ml ANS promoted the accumulation of polypeptides detected by a UFM1-specific antibody of sizes corresponding to UFMylated RPL26 (Figure S1A). The intensity of the bands decreased at higher ANS concentrations, which is in line with a more pervasive translation arrest and consequent decrease of ribosome collision but remained constant upon treatment for up to 1 h (Figure S1B). HeLa cells treated with 50 ng/ml ANS for 1 h were then lysed in denaturing buffer containing 20 mM NEM and 10 mM iodoacetamide to inhibit the activity of Ub/UbL deconjugases and MYC-tag immunoprecipitates were analyzed by immunoblots. Three bands of sizes corresponding to RPL26 conjugated to one, two, and three UFM1 moieties were readily detected by the UFM1-specific antibody in the MYC immunoprecipitates of ANS-treated control and BPLF1^C61A^ expressing cells. The two lower bands correspond in size to the previously described RPL26 Lys134 and Lys132 UFM1 adducts [24], while the third band may indicate poly-UFMylation at either site or the presence of a third UFMylated Lys residue. The bands were virtually absent in the lysates of cells expressing active BPLF1, suggesting that the vDUB efficiently blocks UFMylation (Figure 2A). To validate this finding and to confirm that the UFMylation of RPL26 occurs on ribosomes, which would exclude artifacts due to incomplete incorporation of the overexpressed RPL26-MYC, cytosol and ER-enriched fraction of lysates from control and ANS-treated cells co-transfected with FLAG-ev, -BPLF1 or -BPLF1^C61A^ and RPL26-MYC were probed with the RPL26 antibody (Figure 2B). This experimental setup allowed us to distinguish between endogenous RPL26 present in both transfected and non-transfected cells (Figure 2B black arrows) and the slightly slower migrating MYC-tagged RPL26 that is only expressed in the transfected cells (Figure 2B, red arrows). The fractionation procedure confirmed the previous observation that ANS treatment promotes the UFMylation of RPL26 exclusively on ER-associated ribosomes [12,24] (compare the intensity of endogenous UFMylated species, black arrows, and UFMylated RPL26-MYC, red arrows, in the cytosol and ER fractions of untreated, lanes 2–3, and ANS treated cells lanes 5–6) and verified the virtually complete absence of ANS-induced UFMylation in ribosome enriched fraction of cells coexpressing catalytically active BPLF1, whereas the mutant BPLF1^C61A^ had not effect (Figure 2B, compare lanes 6, 9 and 12, red arrows). The persistence of some endogenous UFMylated RPL26 in the ER fraction of ANS-treated BPLF1-expressing cells (compare lanes 6 and 9, black arrows) likely reflects the presence of a population of non-transfected cells. Interestingly, bands of size corresponding to endogenous mono and di-UFMylated endogenous RPL26 were detected in the cytosolic fractions of untreated cells (lanes 1 and 2, black arrows) and their intensity was not affected by ANS treatment or BPLF1 expression (compare lanes 2, 5, 7, and 11, black arrows), suggesting that RPL26 UFMylation occurs physiologically in the absence of overt ribosomal stress and that UFMylated RPL26 is then released to the cytosol where it is not targeted by BPLF1.

**Figure 3.**
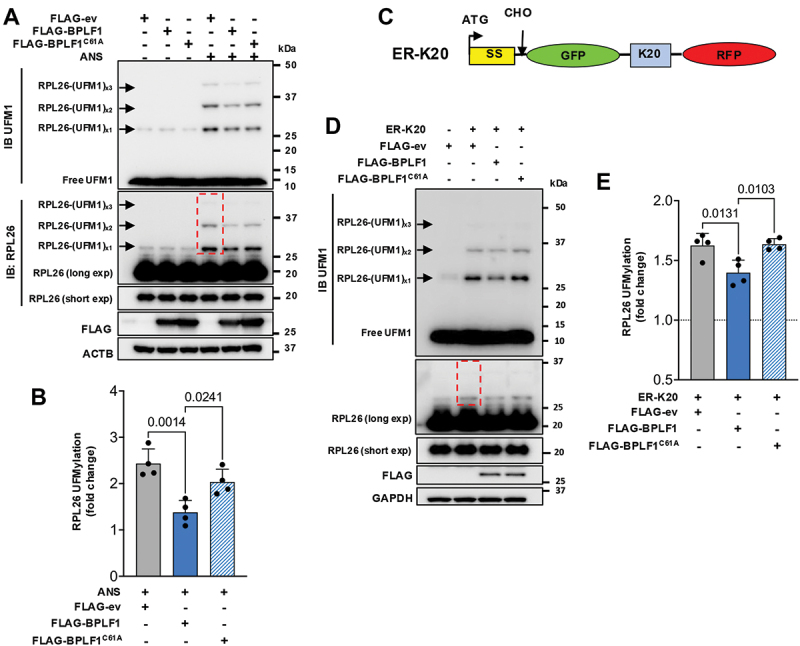
BPLF1 inhibits the UFMylation of endogenous RPL26. (A) FLAG-ev, -BPLF1 or -BPLF1^C61A^ transfected HeLa cells were cultured for 24 h and then treated with 50 ng/ml ANS for 1 h before lysis in buffer containing NEM and iodoacetamide to inhibit DUB activity. High molecular weight species corresponding to the size of mono- di- and tri-UFMylated RPL26 were detected in immunoblots probed with antibodies specific for RPL26, and bands of the same size were detected after stripping and re-probing the blot with the UFM1 antibody. Representative blots from one out of four independent experiments are shown in the figure. (B) densitometry quantification of the intensity of the UFMylated species. A red dotted box indicates the area included in the densitometry scan. The intensity was normalized to the intensity of the RPL26 band in short exposure of the same blots. The mean ± SD fold increase in ANS treated relative to untreated cells in four independent experiments is shown. Significance was calculated by unpaired two-tailed Student’s t-test. (C) schematic illustration of the modified ER-RQC reporter. The reporter expresses in-frame an N-terminal ER-targeting signal sequence followed by an N-glycosylation site, GFP, and a stretch of lys residues encoded by AAA codons (K20) and RFP. Stalling of the ribosome at the poly(A) sequence traps the nascent ER-inserted polypeptide in the translocon. (D) BPLF1 inhibits the UFMylation of endogenous RPL26 induced by the ribosome stall-inducing reporter. HEK293T cells were cotransfected with FLAG-ev, -BPLF1 or -BPLF1^C61A,^ and the modified ER-K20 reporter and immunoblots of cells harvested after 24 h were probed with the indicated antibodies. Immunoblots from one representative experiment out of four are shown in the figure. (E) densitometry quantification of the UFMylated proteins in four independent experiments. A red dotted box indicates the area included in the densitometry scan. Fold change was calculated relative to vector-transfected cells after normalization to the intensity of the RPL26 band in short exposure of the same blots. Statistical analysis was performed using an unpaired two-tailed Student’s t-test.

To further validate the inhibitory effect of BPLF1, the UFMylation of endogenous RPL26 was assayed in immunoblots of untreated and ANS-treated control, and FLAG-ev, -BPLF1 or -BPLF1^C61A^ transfected HEK293T cells. Three additional bands of size corresponding to mono-, di- and tri-UFMylated RPL26 were detected by the RPL26 specific antibody in the ANS-treated controls (Figure 3A upper panel), and bands of identical sizes were detected by re-probing the blots with antibodies to UFM1, supporting their identification as UFMylated RPL26 species (Figure 3A, lower panel). Of note, a band of size corresponding to mono-UFMylated RPL26 was often detected by the UFM1-specific antibody in the untreated cells, which is consistent with the presence of UFMylated RPL26 in the cytosolic fraction of untreated cells (see Figure 2B). The intensity of the UFMylated species was significantly decreased in cells expressing catalytically active BPLF1, while the catalytic mutant had no consistent effect (Figure 3B). The apparently weaker effect of the vDUB on the UFMylation of endogenous RPL26 compared to the virtually complete inhibition observed following MYC-immunoprecipitation of lysates from RPL26-MYC-FLAG-BPLF1 cotransfected cells is likely explained by a relatively low efficiency of transfection, resulting in a high background of UFMylation in non-transfected cells. This effect is further amplified in the RPL26 blots by the relatively low abundance of collided ribosomes that stall at the ER compared to the total ribosome pool, which required overexposure of the blot to detect the UFMylated species. To further assess whether BPLF1 inhibits the UFMylation of RPL26 on ribosomes that stall during co-translational ER translocation, we used a modified version of the ER-targeted reporter plasmid ER-K20 [26]. The modified reporter expresses, in-frame, an HLA class I ER-localization signal followed by an H-glycosylation sequence and GFP fused to a poly (A) encoded twenty poly-lysine tract (K20) and RFP (Figure 3C). Significant inhibition of RPL26 UFMylation was observed in BPLF1 transfected HEK293T cells expressing the stalling reporter (Figure 3D). In contrast, the mutant BPLF1^C61A^ had no significant effect (Figure 3E), which further supports the conclusion that the vDUB affects the UFMylation of RPL26 on ER-associated stalled ribosomes.

### BPLF1 is not a deUfmylase and does not interfere with the assembly and activity of the UFM1 ligase

**Figure 4.**
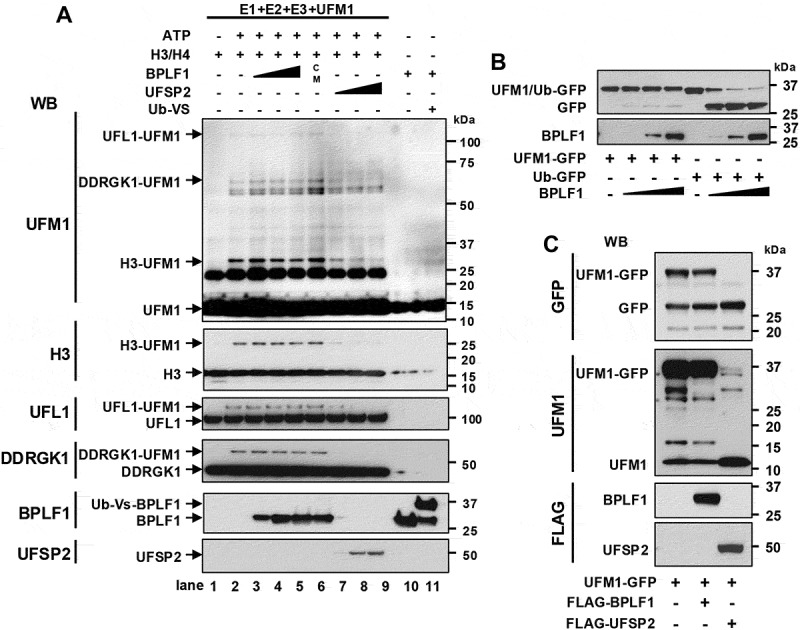
BPLF1 does not have deUfmylase activity. (A) Representative immunoblots of an *in vitro* UFMylation reaction performed in the presence or absence of BPLF1 or UFSP2. UFMylation reactions were performed in the presence of purified components of conjugation cascade: 0.25 μM recombinant His-UBA5 (E1), 5 μM GST-UFC1 (E2), 1 μM His-UFL1/Strep-DDRGK1 (E3), 0.5 μM of H3/H4 complex (substrate), and 10 μM His-UFM1 in the absence (lane 1) or presence of 5 mM ATP (lanes 2–9). Where indicated increasing concentrations of recombinant BPLF1 (lanes 3–5; 0.3, 0.75, and 1.5 μM, respectively), His-BPLF1^C61A^ (CM, lane 6; 1.5 μM) or His-UFSP2 (lane 7–9; 0.03, 0.075, and 0.15 μM, respectively) were included in the reaction. The DUB activity of recombinant BPLF1 was confirmed by labeling 1.5 μM His-BPLF1 with 1 μM of the functional probe HA-Ubiquitin-vinyl-sulphone (Ub-Vs) that forms covalent adducts with the catalytic cys residue (lane 10–11). All reactions were incubated at 37°C for 90 min and analyzed in immunoblots using the indicated antibodies. UFMylated H3/H4, UFL1, and DDRGK1 were detected when the reaction was performed in the presence of ATP (lane 2). The addition of increasing amounts of BPLF1 or BPLF1^C61A^ had no effect (lanes 3–6), while efficient de-UFMylation was induced by minute amounts of UFSP2 (lanes 7–9). The experiment was repeated twice with comparable results. UFM1 dimers were detected by the UFM1 antibody as a strong band of approximately 20 kDa. (B) recombinant BPLF1 does not cleave a UFM1-GFP reporter. Increasing amounts of purified recombinant BPLF1 (0.3, 0.75, 1.5 µM) were mixed with the UFM1-GFP or Ub-GFP reporters (0.3 µM) in reaction buffer and incubated for 1 h at 37°C followed by immunoblot analysis. One representative experiment out of three is shown in the figure. (C) Representative immunoblots illustrating the failure of BPLF1 to cleave a UFM1-GFP reporter in cells. U2OS-*UFSP2*-knockout cells were cotransfected with a UFM1-GFP fusion protein-expressing plasmids and plasmids expressing BPLF1 or the cellular deUfmylase UFSP2. The production of free GFP and UFM1 was monitored in immunoblots probed with specific antibodies. The reporter was cleaved only in cells expressing UFSP2. One representative experiment out of two is shown.

In the next set of experiments, we explored the mechanism by which BPLF1 regulates UFMylation. We first asked whether, besides the known specificity for ubiquitin and Nedd8 conjugates [56], the vDUB might also have deUFMylase activity. To this end, recombinant BPLF1 and the cellular deUFMylase UFSP2 were compared for their capacity to counteract the UFMylation of the known UFMylation substrate histone H3/H4 [57] by purified components of the UFMylation cascade. UFMylated H3 was readily detected in western blots of the UFMylation reaction performed in the presence of ATP (Figure 4A). The addition of recombinant catalytically active BPLF1 or mutant BPLF1^C61A^ (indicated in Figure 4A as C/M) had no appreciable effect, whereas UFMylation was abolished by the addition of the cellular deUFMylase UFSP2. Similar results were obtained when the hydrolysis of recombinant Ub-GFP and UFM1-GFP fusion proteins that carry the recognition sites of Ub/UFM1-specific deconjugases [58] was assayed in the presence of increasing amounts of recombinant BPLF1 (Figure 4B). While Ub-GFP was efficiently cleaved at the lowest BPLF1 concentration, UFM1-GFP was only marginally affected even by the highest enzyme:substrate ratio. To confirm that the UFM1-GFP reporter is a bona fide deUFMylase substrate and explore the possibility that critical co-factors may be missing in the *in vitro* reaction, eukaryotic expression vectors were cotransfected with FLAG-BPLF1 or FLAG-UFSP2 in a HEK-*UFSP2*-knockout cell line (Figure S2A). Notably, constitutive levels of RPL26 UFMylation were observed in the HEK-*UFSP2*-knockout cell line. This UFMylation was abolished by the reconstitution of UFSP2 but was not affected by the overexpression of catalytically active or inactive BPLF1 (Figure S2B), further substantiating the finding that the vDUB does not affect UFMylation events occurring in the absence of explicit ribosomal stress (Figure S2B). Furthermore, the UFM1-GFP fusion protein was efficiently cleaved in HEK-*UFSP2*-knockout cells upon reconstitution of UFSP2, resulting in the accumulation of free GFP and UFM1 (Figure 4C), whereas the transfection of BPLF1 had no effect. Thus, we concluded that BPLF1 does not have deUFMylase activity, and the effects observed upon the induction of ribosomal stress are not due to deUFMylation.

**Figure 5.**
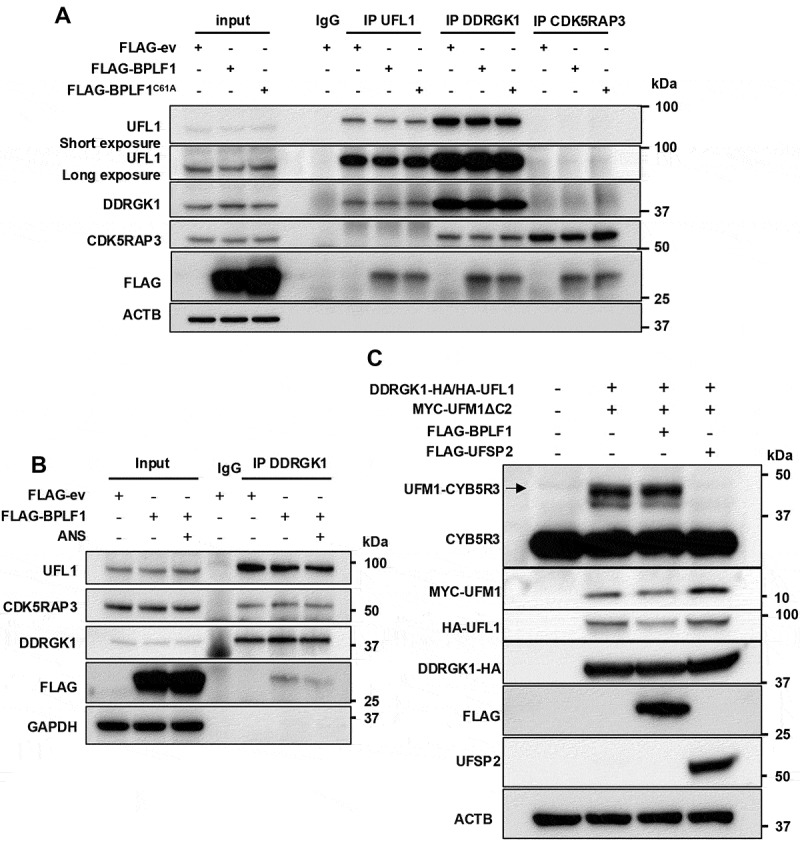
BPLF1 does not affect the assembly and activity of the UFL1 ligase complex. (A) reciprocal co-immunoprecipitation assays illustrate the failure of BPLF1 to affect the interaction of UFL1 with DDRGK1 and CDK5RAP3 in untreated cells. Lysates of HEK293T transfected with FLAG-ev, -BPLF1 or -BPLF1^C61A^ were divided into equal aliquots, and co-immunoprecipitation was carried out with the indicated antibodies. Comparable levels of catalytic active and mutant BPLF1 were detected by the FLAG antibody in the UFL1, DDRGK1, and CDK5RAP3 immunoprecipitates, but their presence did not affect the co-immunoprecipitation of the ligase components. One representative experiment out of three is shown in the figure. (B) BPLF1 does not affect the assembly of the ligase in ANS-treated cells. HEK293T were transfected with FLAG-ev or -BPLF1, and one aliquot of the FLAG-BPLF1 transfected cells was treated with 50 ng/ml ANS for 30 min before harvesting. Immunoprecipitates of endogenous DDRGK1 were probed with the indicated antibodies. Immunoblots from one out of two independent experiments are shown. (C) BPLF1 does not affect the activity of the UFL1 ligase. The UFMylation of CYB5R3 was induced in HEK293-*UFSP2*-knockout cells by transfection of ha-tagged UFL1 and DDRGK1 and MYC-tagged active UFM1 (UFM1ΔC2) with or without co-transfection of FLAG-BPLF1 or -UFSP2. Representative immunoblots from one of two independent experiments are shown.

The ER-associated UFM1 ligase is a trimolecular complex formed by the recruitment of UFL1 and CDK5RAP3 to the adaptor protein DDRGK1. The interaction of UFL1 with DDRGK1 greatly enhances ligase activity, while CDK5RAP3 regulates substrate recognition [30,31] and inhibits the activity of the ligase in *in vitro* UFMylation assay [30]. Given the interaction of BPLF1 with UFL1, we explored the possibility that the vDUB may inhibit UFMylation by interfering with the assembly of the trimolecular complex. To this end, cross-immunoprecipitation of the endogenous proteins was performed in HEK293T cells transfected with FLAG-ev, -BPLF1 or -BPLF1^C61A^. In accordance with previous reports where the interactions were assayed in cells transfected with tagged versions of the proteins [31], both UFL1 and CDK5RAP3 were detected in the immunoprecipitates of endogenous DDRGK1 (Figure 5A). However, only DDRGK1 was detected in the UFL1 immunoprecipitates, and endogenous CDK5RAP3 did not coprecipitate UFL1 or DDRGK1, which may be due to weak interactions or blocking of the binding sites by the specific antibodies used for immunoprecipitation of the endogenous proteins. The efficiency of co-precipitation was not affected by the expression of catalytically active or inactive BPLF1 (Figure 5A), even when the amount of expressed viral protein was enhanced by transfecting an increasing amount of the plasmid (Figure S3). Furthermore, similar levels of UFL1 and CDK5RAP3 co-immunoprecipitation with DDRGK1 were observed in untreated and ANS-treated cells, independent of the presence of BPLF1 (Figure 5B), confirming that the vDUB does not interfere with the assembly of the complex. This conclusion was further substantiated by the finding that overexpressed BPLF1 did not affect the UFMylation of CYB5R3 in HEK-*UFSP2*-knockout cells cotransfected with plasmids expressing HA-UFL1 and DDRGK1 and MYC-UFM1ΔC2 (Figure 5C), whereas UFMylation was virtually abolished by the reconstitution of UFSP2.

### BPLF1 counteracts a ubiquitination event required for RPL26 UFMylation

**Figure 6.**
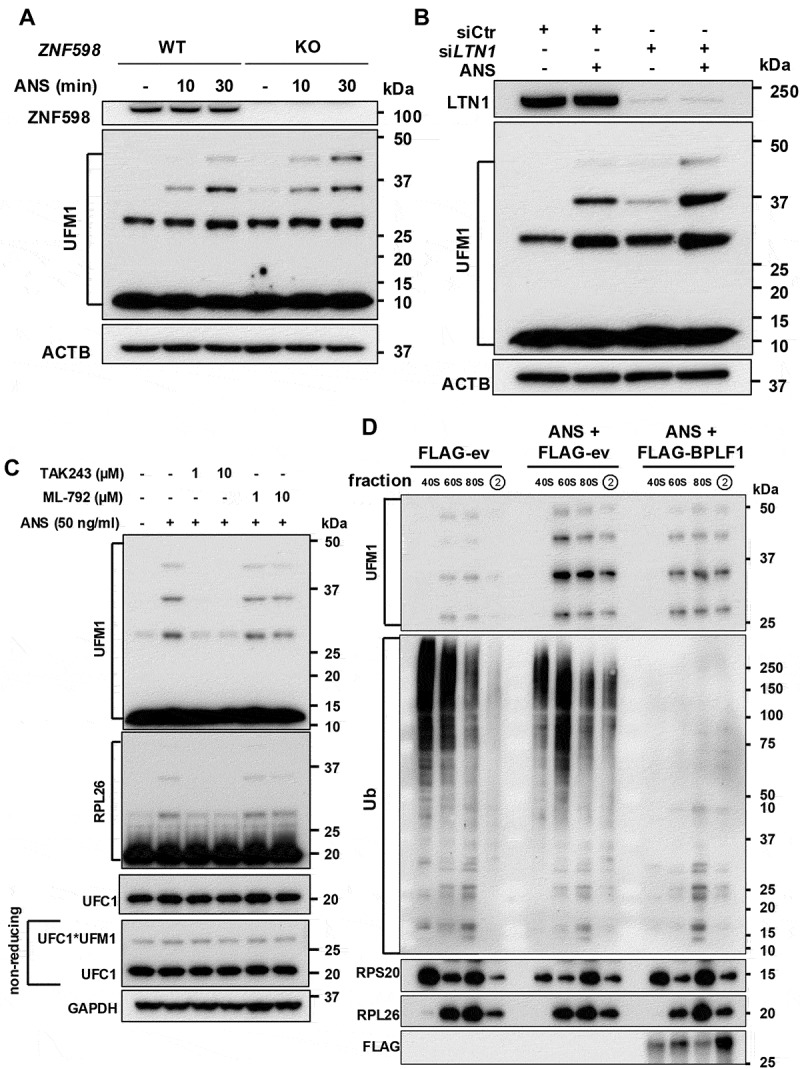
BPLF1 counteracts a ZNF598- and LTN1-independent ubiquitination event required for RPL26 UFMylation. (A) the activity of the ZNF598 ligase is not required for UFMylation of RPL26 in ANS-treated cells. Control (WT) and HEK293T-*ZNF598*-KO cells were treated with 50 ng/ml ANS for the indicated times before analysis of RPL26 UFMylation. Immunoblots from one representative experiment out of three are shown. (B) the activity of the LTN1 ligase is not required for UFMylation of RPL26 in ANS-treated cells. HEK293T cells were transfected with a control scrambled siRNA or a previously characterized LTN1 siRNA for 72 h before treatment with 50 ng/ml ANS for 30 min and analysis of RPL26 UFMylation. Immunoblots from one representative experiment out of two are shown. (C) the inhibition of de novo ubiquitination by pretreatment with a ubiquitin E1 small molecule inhibitor prevents RPL26 UFMylation in ANS-treated cells. HEK293T cells were pretreated with the indicated concentrations of TAK243 or the SUMO E1 inhibitor ML-792 before ANS treatment. UFC1*UFM1 thioesters were detected by running the same lysates without reducing agents. Immunoblots from one representative experiment out of three are shown. (D) cleared lysates of HEK293T transfected with FLAG-ev or -BPLF1 and treated with 50 ng/ml ANS for 20 min were fractionated by centrifugation over a 5–50% sucrose gradient. Fractions enriched in 40S, 60S, and 80S ribosomes and the first polysome fraction were identified (Figure S5) and analyzed by immunoblotting using the indicated antibodies. Immunoblots from one of two independent experiments showing comparable results are shown.

The finding that the vDUB is neither a deUFMylase nor affects the activity of the UFM1 ligase suggests that it may counteract a ubiquitination event required for RPL26 UFMylation. We have previously shown that BPLF1 counteracts the activity of ZNF598 and LTN1 and stabilizes cytosolic RQC substrates [44]. The ZNF598 ligase initiates the RQC by ubiquitinating RPS10 and RPS20 on collided ribosomes, which recruits the RQC-trigger (RQT) complex that splits the ribosome and triggers the LTN1-dependent ubiquitination of the aberrant translation product that remains associated with the 60S [59,60]. To investigate whether inhibition of these ubiquitination events might explain the effect of the DUB on RPL26 UFMylation, the efficiency of RPL26 UFMylation was compared in the HEK293T cell line and sublines carrying CRISPR-Cas9 mediated knockout of *ZNF598* or siRNA mediated knockdown of *LTN1*. Treatment for 30 min with 50 ng/ml ANS efficiently triggered the UFMylation of RPL26 in *ZNF598*-KO cells (Figure 6A). Thus, RPL26 UFMylation does not require the sensing of collided ribosomes or the ubiquitination of 40S proteins by ZNF598, and the subsequent ribosome splitting. Interestingly, the UFMylation of RPL26 appeared to be enhanced when the degradation of the arrested polypeptide was inhibited by LTN1 knockdown in the presence of proficient collision sensing and ribosome splitting (Figure 6B). This finding aligns with the notion that the cytosolic RQC relieves the stress caused by the aberrant translation of ER-targeted proteins [8,12].

The above findings suggest that a previously unrecognized ubiquitination event may regulate the UFMylation of RPL26. To explore this possibility, de novo ubiquitination was inhibited by treating HEK293T cells with increasing concentrations of the specific ubiquitin-activating enzyme inhibitor TAK243 for 3 h before induction of UFMylation by treatment with ANS. As a control, the cells were pretreated with the SUMO-activating enzyme inhibitor ML792. Immunoblots probed with antibodies to ubiquitin, or SUMO confirmed the selective loss of ubiquitination in cells treated with TAK243 and the loss of SUMOylation in cells treated with ML792 (Figure S4). The increase of SUMOylated proteins in cells treated with TAK243 is likely explained by inhibition of the ubiquitin-dependent degradation of SUMOylated substrates. The treatment with TAK243 abolished RPL26 UFMylation in ANS-treated cells, whereas treatment with ML792 had no effect (Figure 6C). The effect of TAK243 cannot be ascribed to cross-reactivity with the UFM1-activating enzyme UBA5 since comparable levels of UFC1*UFM1 thioesters were detected in untreated and treated cells when the samples were run in non-reducing conditions (Figure 6C).

We then reasoned that the regulatory ubiquitination could occur near the UFMylation site, for example, on adjacent ribosome proteins or components of the targeting and translocation machinery that guide the nascent peptide to the ER. To explore this possibility, ribosomes were isolated by sucrose gradient centrifugation from lysates of untreated FLAG-ev transfected cells and cells treated with ANS in the presence or absence of catalytic active BPLF1 (Figure S5). The fractionation procedure was validated by probing immunoblots with antibodies specific for RPS20 and RPL26 to assess the enrichment of 40S, 60S, 80S ribosomes and polysomes (Figure S5B). Interestingly, while most of the transfected BPLF1 was recovered in the early ribosome-depleted fractions, the vDUB was also present in all ribosome fractions but was relatively depleted in the 40S, 60S, and 80S fractions and enriched in the polysome fractions (Figure S5B), pointing to the preferential association with translating ribosome. Analysis of the UFMylation and ubiquitination of the peak 40S, 60S, 80S, and first polysome fractions, which in ANS-treated cells is likely to contain collided disomes, confirmed the low levels of RPL26 UFMylation in untreated cells and the increase upon ANS treatment. Of note, although prevalent in the 60S fraction, RPL26 UFMylation was observed in the 80S and first polysome fractions, which corroborates its independence of ribosome splitting, as suggested by the persistence in *ZNF598*-KO cells (Figure 6A). As expected, the ANS-induced UFMylation of RPL26 was decreased in BPLF1-expressing cells (Figure 6D), and this correlated with a striking decrease in ubiquitination in all ribosome fractions. The relatively high ubiquitination levels detected in untreated cells are likely explained by the presence of both ribosome and ribosome-associated proteins whose activity is regulated by ubiquitination independently of translational stress. Importantly, while high molecular weight polyubiquitination was depleted in all ribosome-containing fractions, some ubiquitinated species, particularly in the low molecular weight range, appeared to be either unaffected or slightly increased, which speaks against a nonspecific global deubiquitination effect due to overexpression of the transfected vDUB. Collectively, these findings support the conclusion that BPLF1 inhibits UFMylation by counteracting a constitutive or ribosome-stress-induced ubiquitination event. The ligase and putative substrate(s) of this previously unrecognized regulatory step of the ER-RQC are currently unknown.

### BPLF1 stabilizes an ER-RQC substrate, inhibits reticulophagy, and activates ER-stress responses

**Figure 7.**
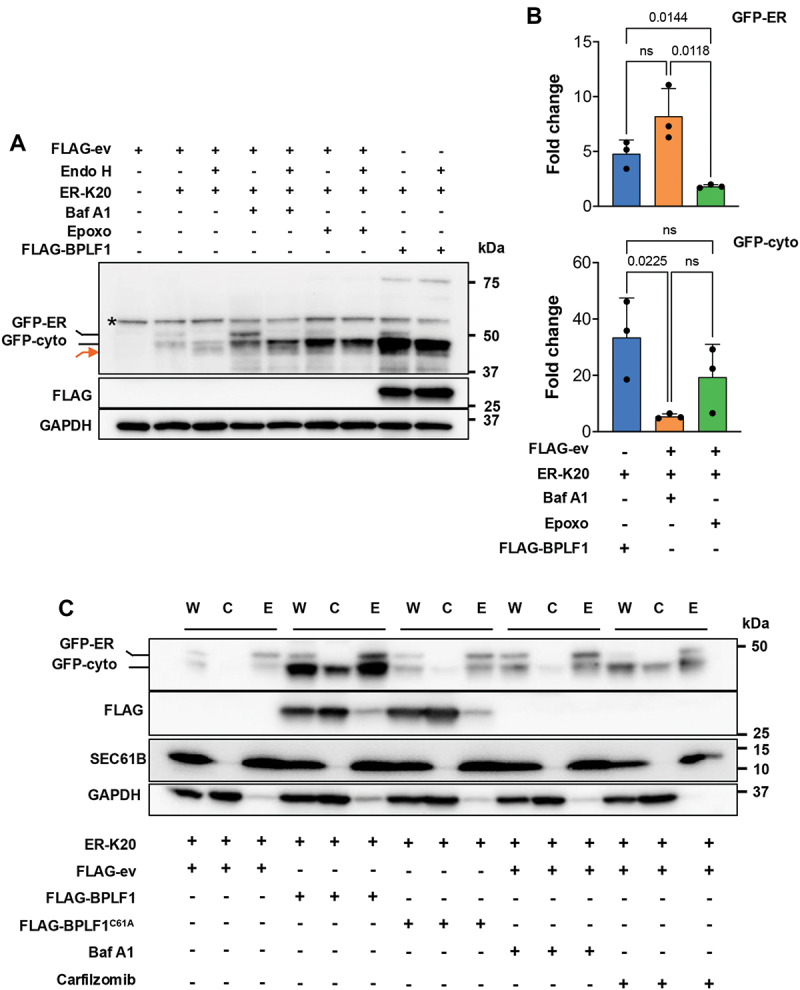
BPLF1 rescues ER-RQC substrates in the cytosol and the ER. (A) BPLF1 stabilizes an ER-RQC substrate. HEK293T cotransfected with the ER-K20 reporter and FLAG-ev, -BPLF1 or -BPLF1^C61A^ were cultured for 24 h. Aliquots of FLAG-ev and ER-K20 transfected cells were treated with 100 nM Baf A1 or 100 nM epoxomicin (epoxo) overnight before harvesting. The de-glycosylated GFP-ER product is indicated by a red arrow. Immunoblots from one representative experiment out of three are shown in the figure. A nonspecific band detected by the anti-GFP antibody is indicated by an asterisk (*). (B) Densitometry quantification of the GFP bands. Fold increase was calculated as the ratio between the band’s intensity in treated cells versus cells transfected with the ER-K20 reporter alone. The average fold increase in three independent experiments is shown. Statistical analysis was performed using an unpaired two-tailed Student’s t-test. (C) BPLF1 stabilizes the ER-RQC substrate in the cytosol and ER. HEK293T cells were transfected and treated as described in (A), except that carfilzomib was used instead of epoxomicin to inhibit proteasome activity. Unfractionated (W), cytosolic (C), and ER-membrane (E) fractions were produced as described in the methods section. Immunoblots from one out of three independent experiments are shown in the figure.

Compelling evidence links the UFMylation of RPL26 with the ER-RQC-dependent degradation of translocon-trapped polypeptides via either proteasome or lysosome-dependent mechanisms [24,26]. To assess whether BPLF1 affects this process, HEK293T cells were cotransfected with the FLAG-ev, -BPLF1 or -BPLF1^C61A^ plasmids and the ER-K20 reporter, and the abundance of the translated products was assessed in immunoblots probed with the GFP antibody. As controls for lysosome and proteasome-dependent degradation, FLAG-ev cotransfected cells were treated overnight with bafilomycin A_1_ (Baf A1) or either epoxomicin (Epoxo) or carfilzomib to inhibit proteasome activity. To assess the subcellular localization of the translated products, the cell lysates were treated with Endo H, which cleaves N-linked glycans that are cotranslationally attached to nascent peptides in the ER lumen [61,62]. Two faint GFP bands of approximately 50 and 48 kDa were detected in FLAG-ev transfected HEK293T cells (Figure 7A). The lower band corresponds in size to a precursor peptide arrested at the poly (A) sequence that is non-glycosylated and contains the signal peptide. This, together with the resistance to Endo H treatment, indicates that the peptide is either released in the cytosol or if it remains associated with the translocon, accumulates at the cytosolic face of the ER. The sensitivity to Endo H treatment identifies the upper band as an ER-localized glycosylated peptide that, based on the migration shift induced by the treatment, lacks the signal peptide (Figure 7A, red arrow). In line with the notion that lumenal ER-RQC substrates are targeted for lysosome-dependent degradation, the 50-kDa species accumulated in cells treated with Baf A1, whereas epoxomicin, carfilzomib and Baf A1 independently promoted the accumulation of the non-glycosylated precursor peptide (Figure 7A,B). Subcellular fractionation confirmed the presence of the precursor peptide in both cytosol, where it was exclusively targeted for proteasomal degradation, and the ER membrane where it was stabilized by treatment with either Baf A1 or carfilzomib. In line with the stabilization by Baf A1, the glycosylated peptide was exclusively found in the ER fraction (Figure 7C). The glycosylated ER lumenal and non-glycosylated cytosolic or ER-associated species were all stabilized in cells expressing catalytically active BPLF1 (Figure 7A–C). In addition, a 75-kDa polypeptide corresponding to the full-length product was selectively stabilized in BPLF1-expressing cells (Figure 7A), which is consistent with our previous finding that, by inhibiting the RQC, BPLF1 promotes the readthrough of stall-inducing mRNAs [44]. Thus, while inhibition of the RQC ligases explains the stabilization of the precursor peptide in the cytosol, the stabilization of the ER-membrane-associated precursor, and particularly the ER lumenal glycosylated products points to an unexpected effect of the vDUB on lysosome-dependent degradation.

**Figure 8.**
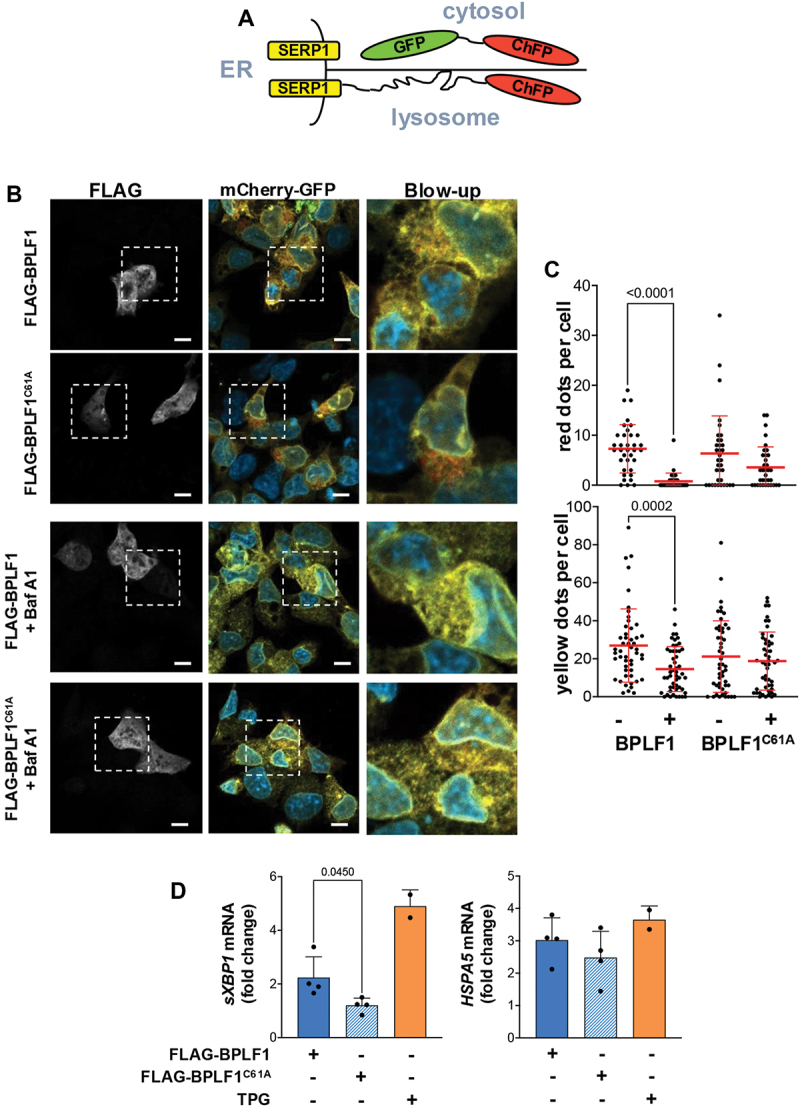
BPLF1 inhibits reticulophagy and triggers ER-stress responses. (A) schematic illustration of the ER-Autophagy Tandem Reporter (EATR). The reporter expresses in-frame the coding sequence of the SERP1 subunit of the ER translocon complex followed by the coding sequences of eGFP and ChFP. Upon ER insertion of the SERP1 domain, eGFP and ChFP face the cytosol and emit equal fluorescence, whereas, due to the selective loss of eGFP fluorescence at low pH, ER-loaded autophagosomes appear as distinct red fluorescent dots. (B) Representative confocal images that illustrate the failure to accumulate red fluorescent dots or yellow fluorescent dots in cells expressing active BPLF1 starved in the absence or presence of Baf A1, respectively. Stable HCT116-EATR cells were transfected with plasmids expressing FLAG-BPLF1 or -BPLF1^C61A^ and then starved overnight in EBSS medium before visualizing the formation of ER-loaded autophagosomes by confocal microscopy. Scale bar: 10 μm. (C) quantification of the number of red (upper panel) and yellow (lower panel) fluorescent dots in BPLF1- or BPLF1^C61A^-positive and -negative cells from the same transfection experiments. The cumulative data from two independent experiments where approximately 50 vDUB-positive and -negative cells scored from the same slide are shown. Significance was calculated using an unpaired two-tailed Student t-test. (D) BPLF1 promotes the accumulation of the ER stress markers spliced *XBP1* mRNA and the transcriptional upregulation of *HSPA5*. FLAG-ev transfected cells treated with thapsigargin during the last 2 h before harvest were included as a reference of ER-stress induction. Quantification of *sXBP1* and *HSPA5* mRNAs in four independent experiments. Fold induction was calculated relative to untreated FLAG-ev transfected cells after normalization to the *GAPDH* housekeeping gene. Significance was calculated using an unpaired two-tailed Student t-test.

The UFMylation of ribosomes and ER-membrane proteins was shown to regulate reticulophagy that participates in the clearance of ER-RQC substrates [25,27,28]. To test whether the effect of BPLF1 on ribosome UFMylation impacts reticulophagy, we produced a subline of HCT116 expressing a Dox-regulated reticulophagy dual fluorescence reporter constructed by in-frame fusion of the coding sequences of the SERP1/RAMP4 subunit of the ER translocon, enhanced GFP (eGFP), and mCherry fluorescent protein (ChFP) [63] (Figures 8A and Figure S6). Upon ER insertion of the SERP1 domain, GFP and ChFP face the cytosol and emit equal fluorescence, whereas, due to the selective loss of GFP fluorescence at low pH, ER-loaded autolysosomes appear as distinct red, fluorescent dots (Figure S6B). As expected, the inhibition of autophagosome acidification by treatment with Baf A1 resulted in the accumulation of yellow vesicles where both GFP and mCherry fluorescence were preserved (Figure S6C). To assess the effect of BPLF1 on this process, the reporter cell line was transfected with plasmids expressing FLAG-BPLF1 or -BPLF1^C61A^ and cultured for 24 h in the presence of Dox before being starved overnight in EBSS medium followed by visualization of autophagosomes and autolysosomes by confocal microscopy. Of note, starvation-induced reticulophagy was previously shown to be dependent on the activity of the UFL1 ligase [25,28] and correlated with the UFMylation of RPL26. Red cargo-loaded autolysosomes were readily detected in cells expressing the BPLF1^C61A^ mutant, whereas only a diffuse yellow fluorescence was observed in most cells expressing the active BPLF1 (Figure 8B). Quantification of the number of red dots in BPLF1- or BPLF1^C61A^-positive and -negative cells from the same transfection revealed highly significant suppression of reticulophagy in cells expressing the catalytically active vDUB (Figure 8C). To assess whether the formation of autophagosomes was affected, the acidification of autolysosome was inhibited by treatment with Baf A1. Treatment with Baf A1 during starvation promoted the accumulation of yellow dots in cells expressing the mutant BPLF1^C61A^ but not in cells expressing the active enzyme, supporting the conclusion that the vDUB inhibits an early step of the pathway that precedes the formation of autophagosomes.

The impairment of reticulophagy was shown to trigger ER stress responses due to the accumulation of aberrant translation products in the ER lumen [64]. Commonly used markers of ER-stress activation include splicing of the XBP1 precursor mRNA (XBP1s) by the activated stress sensor ERN1/IRE1α, which promotes expression of the XBP1-spliced transcription factor and upregulation of its target genes, including the ER chaperone HSPA5/BiP [65]. To assess whether the inhibition of UFMylation correlates with the induction of ER stress, the expression of *XBP1s* and *HSPA5/BiP* mRNAs was quantified in HEK293T cells transfected with FLAG-ev, -BPLF1 or -BPLF1^C61A^. As a reference for ER stress induction, the cells were treated with 1 µM thapsigargin (TPG) for 2 h before harvesting. The expression of catalytically active BPLF1 was accompanied by significant upregulation of *XBP1s* mRNA compared with the catalytic mutant BPLF1^C61A^ (Figure 8D, left panel). In line with the higher expression of *XBP1s* mRNA, higher levels of *HSPA5* mRNA were usually detected in cells expressing BPLF1 (Figure 8D, right panel). However, due to the relatively small effects, high variability between experiments, and possible confounding effect of a general stress response induced by the transfection procedure, the difference between active and inactive enzymes did not reach statistical significance.

### Physiological levels of BPLF1 inhibit RPL26 UFMylation in EBV-infected cells

**Figure 9.**
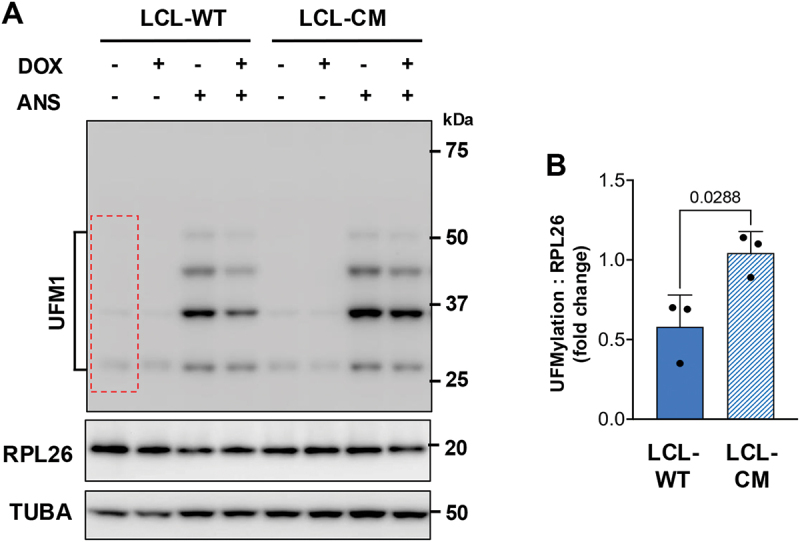
Physiological levels of BPLF1 inhibit RPL26 UFMylation in productively EBV-infected cells. (A) the productive virus cycle was induced in LCLs immortalized with recombinant EBV expressing wild-type (LCL-WT) or catalytic mutant (LCL-CM) BPLF1. After 72 h, equal aliquots of cells were treated with 50 ng/ml ANS for 30 min, followed by the analysis of RPL26 UFMylation by immunoblots. Blots from one representative experiment out of three are shown. (B) densitometry quantification of the UFMylated species in three independent experiments. A red dotted box indicates the area included in the densitometry scan. Data are shown as fold induction of UFMylated RLP26 in untreated versus ANS-treated cells after normalization to the TUBA loading control and total RPL26. The mean ± SD fold change in induced relative to uninduced cells is shown. Significance was calculated using an unpaired two-tailed Student t-test.

To investigate whether physiological levels of the vDUB regulate ribosome UFMylation, the productive virus cycle was induced in a pair of lymphoblastoid cell lines (LCLs) established by immortalization of B lymphocytes with recombinant EBV encoding catalytically active BPLF1 (LCL-WT) or mutant BPLF1^C61A^ (LCL-CM) and carrying a Dox-regulated EBV transactivator BZLF1, which ensures efficient and synchronous induction [46,66] (Figure S7). Repeated attempts to detect changes in the endogenous levels of RPL26 UFMylation during productive infection gave inconsistent results, which is likely due to a relatively low frequency of ER-stalled ribosomes and to the confounding effects of more than eighty different viral proteins that are expressed with different kinetics during the productive virus cycle. To circumvent this technical limitation and to achieve detectable levels of ribosome RPL26 UFMylation, control uninduced and cells induced by culture in the presence of Dox for 72 h were treated with 50 ng/ml ANS for 30 min before analysis of RPL26 UFMylation by Western blot. Comparable levels of RPL26 UFMylation were detected upon ANS treatment in non-induced cells independent of the presence of wild-type or BPLF1-mutant virus, confirming that the detection of ribosome stalling and UFMylation machinery are functional in this cell type. Similar levels of ANS-induced RPL26 UFMylation were also observed when the productive cycle was induced in the LCL-CM cells while significantly reduced levels were reproducibly observed in cells expressing catalytically active BPLF1 (Figure 9A,B). Thus, the amount of active enzyme physiologically expressed during the productive virus cycle appears to be sufficient to inhibit the UFMylation of RPL26 on stalled ribosomes.

## Discussion

The importance of ribosome UFMylation in regulating the ER-RQC and reticulophagy, which safeguards the translation of secretory and membrane proteins by promoting the degradation of faulty polypeptides generated during co-translational ER insertion, is firmly established. However, the molecular events that trigger UFMylation and how this guides the disposal of defective translation products remain poorly understood. We found that the ubiquitin deconjugase encoded by the human oncogenic herpesvirus EBV prevents the UFMylation of ribosomes that stall at the ER and inhibit reticulophagy. Unexpectedly, the inhibitory effect involved the abrogation of a ubiquitination event that appears to be required to enable UFMylation and is distinct from the ubiquitination of RPS10 and RPS20 that is triggered by ribosome stalling and collision. The capacity of the vDUB to protect a model ER-RQC substrate from both proteasome and reticulophagy-dependent degradation provides insight into the multiple fates of translocon-trapped polypeptides.

We have previously reported that the vDUB encoded in the N-terminal domain of the large tegument protein of EBV counteracts the activity of the RQC ligases ZNF598 and LTN1 [44]. The presence in the BPLF1 interactome of the UFM1 ligase UFL1 and several putative UFMylation substrates, including the major UFMylation substrate RPL26, hinted at a more general role of the vDUB in regulating RQC responses. Indeed, a reanalysis of our mass spectrometry data under less stringent conditions placed the vDUB in a cellular environment that includes, in addition to translating ribosomes, protein complexes involved in the recognition and delivery of nascent polypeptides to ER translocation (Figure 1A). Direct interaction with UFL1 (Figure 1C) may recruit the vDUB to this environment, but other interactions, either in the context of the trimolecular ligase complex, other ER membrane proteins, or the ER-associated ribosomes, could also play a role. Indeed, we found that catalytically active and inactive BPLF1 were coprecipitated by endogenously expressed UFL1, DDRGK1, and CDK5RAP3. Thus, BPLF1 May interact independently with each ligase component or be brought to the complex by additional binding partners. It is noteworthy that while structural and functional analysis obtained with purified recombinant proteins or in cells transfected with tagged proteins conclusively identify the ER-associated active ligase as a trimolecular complex of UFL1 with DDRGK1 and CDK5RAP3, the assembly of the endogenously expressed proteins in cells has not been explored.

Independently of the primary recruiting partner (s), the presence of BPLF1 in protein complexes, including the UFM1 ligase and ER-associated UFMylation substrates, pointed to the vDUB as a possible regulator of UFMylation and UFMylation-regulated processes. Indeed, catalytically active BPLF1 effectively counteracted the UFMylation of cotransfected MYC-tagged RPL26 in cells treated with the translation elongation inhibitor ANS and inhibited the UFMylation of endogenous RPL26 in cells expressing an ER-RQC reporter encoding a ribosome stall-inducing poly(A) sequence (Figures 2 and 3). Subcellular fractionation experiments confirmed the previous finding that only ER-membrane-associated ribosomes are UFMylated in ANS-treated cells [12] and showed that BPLF1 cofractionated with the ER membrane (Figure 2B). Unexpectedly, two bands corresponding to mono and di-UFMylated endogenous RPL26 were detected in the cytosolic fractions of untreated cells, and their intensity was not affected by ANS treatment or BPLF1 expression. The effective depletion of SEC61B makes the contamination by ER-associated ribosome unlikely, which implies that RPL26 UFMylation occurs constitutively in cells in the absence of overt translational stress and leads to the release of UFMylated ribosome particles in the cytosol. This possibility is supported by recent findings that illustrate a pivotal role of UFMylation in the post-termination release of the large ribosomal subunit from the ER membrane [67,68]. Interestingly, the EBV-encoded G-proteins-coupled receptor BILF1 was recently identified as a UFL1 interactor [69]. BILF1 was shown to redirect the activity of the ligase to MAVS (mitochondrial antiviral signaling protein), which promoted lysosome-dependent MAVS degradation and inhibited the activation of antiviral inflammatory responses. Like BPLF1, BILF1 is expressed during the productive virus cycle, suggesting that the two viral proteins could synergistically inhibit UFMylation-dependent cellular antiviral responses.

Our efforts to elucidate the mechanism by which BPLF1 inhibits UFMylation have yielded an unexpected insight into the requirement of ubiquitination for RPL26 UFMylation. The initial hypothesis that BPLF1 May be the first example of a viral deUFMylase was conclusively disproven by the finding that, unlike the cellular deUFMylase UFSP2, the vDUB did not hydrolyze UFM1 adducts in *in vitro* UFMylation/deUFMylation reactions or in cells expressing an UFM1-GFP fusion protein. We have also excluded the possibility that overexpression of the viral protein might impact the assembly (Figure 5A,B) or activity of the UFM1 ligase, as confirmed by the efficient UFMylation of CYB5R3 (Figure 5C), an ER membrane-resident UFMylation substrate found in the BPLF1 interactome that was recently shown to regulate UFMylation-dependent reticulophagy [28]. Given the capacity of BPLF1 to counteract the activity of the ZNF598 ubiquitin ligase, we then hypothesized that, like for the cytosolic RQC, the sensing of ribosome collision and subsequent ubiquitination of 40S ribosome proteins may be required to trigger the ER-RQC and promote RPL26 UFMylation. This possibility was again disproven by the finding that the RPL26 UFMylation was not abolished by the knockout of ZNF598 (Figure 6A). Thus, RPL26 UFMylation occurs independently of the recognition and ubiquitination of collided ribosomes by ZNF598, pointing to a different triggering context. Based on the observation that the UFMylation of RPL26 is enhanced by the knockdown of LTN1 (Figure 6B) it is tempting to speculate that UFMylation may be triggered by the sensing of persistent translocon clogging.

The findings that the UFMylation of RPL26 occurs independently of the activity of the known RQC ligases and is inhibited by a deubiquitinase that acts on ER-associated ribosomes suggest that the ubiquitination of protein (s) close to the ribosome peptide exit site and ribosome-translocon junction may serve as a licensing factor. Two lines of evidence support a scenario where the inhibition of RPL26 UFMylation depends on the abrogation of a constitutive or ANS-induced ubiquitination event. First, treatment with the E1 inhibitor TAK243 severely impaired the UFMylation of RPL26 in ANS-treated cells (Figure 6C), which substantiates the enabling role of ubiquitination. In addition, ribosome fractionation by sucrose gradient revealed the presence of BPLF1 in the 40S, 60S, and 80S ribosome fractions and its apparent enrichment in the polysome fractions (Figure S5B), which places the vDUB in close contact with translating ribosomes. The interaction with BPLF1 was accompanied by a dramatically decreased ubiquitination in all ribosomal fractions and a parallel reduction of RPL26 UFMylation (Figure 6D). Although overexpression of the active vDUB may have contributed to the magnitude of the effect, the persistence or even increase of several ubiquitinated species speaks against a nonspecific effect due to global deubiquitination. It is noteworthy that the BPLF1 interactome includes, in addition to ribosome proteins, members of several protein complexes, such as the SEC61 translocon, the signal recognition particle (SRP), the ER-membrane proteins complex (EMC), and the OST complex (Figure 1A) that interact with translating ribosomes and regulate the dynamics of ER-association and peptide translocation [70,71]. Whether and how ubiquitination may regulate the activity of these complexes has not been systematically investigated. Thus, a detailed analysis of the constitutive and stress-induced ubiquitination patterns of ER-associated ribosomes and their interacting partners will be required to identify the substrates and effectors of this previously unrecognized step in the regulation of the ER-RQC.

Although the available literature establishes an essential role of RPL26 UFMylation for the disposal of ER-RQC substrates, different scenarios have been proposed for the degradation of the arrested polypeptides. In some cases, degradation by the proteasome was shown to occur in the cytosol following LTN1-dependent ubiquitination and p97/VCP-mediated extraction of the arrested polypeptides from the clogged translocon [12,72,73]. In other cases, the degradation was shown to occur in lysosome following either reticulophagy [27,55] or the chaperone-mediated transport of the peptide to a Golgi exit site [14]. The discrepancies have been attributed to intrinsic properties of the translated product, such as the strength of the ER localization signal and the presence of tightly folded domains [9,12,74]. Using a modified version of the ER-K20 reporter, we have found that catalytically active BPLF1 stabilized both a non-glycosylated precursor peptide that still contains the ER-localization signal and a glycosylated product that lacks the signal sequence and is therefore released in the ER lumen. This conclusion is supported by the effect of Endo-H treatment on the subcellular fraction of cells treated with proteasome inhibitors or Baf A1. The lack of glycosylation indicates that the bulk of the precursor peptide faces the cytosol. Part of it appeared to be released in the cytosol, where it was degraded by the proteasome, but a substantial fraction remained associated with the ER membrane and was stabilized by the inhibition of both proteasome and lysosome-dependent degradation. As expected, the glycosylated lumenal product was only stabilized by treatment with Baf A1. Thus, our findings suggest that the UFMylation of RPL26 concomitantly engages multiple degradation pathways that contribute to the elimination of the arrested peptide. What determines the choice between pathways remains unclear, but the capacity to proficiently engage the translocon and the kinetics of translocon transit, glycosylation, and folding are likely to contribute. Importantly, the capacity of the vDUB to mimic the rescue of the ER lumenal reporter induced by Baf A1 pointed to the involvement of BPLF1 in the regulation of reticulophagy. Indeed, we found that the viral enzyme prevented the trapping of an ER-membrane reporter in autophagic vesicles (Figure 8B,C), which is consistent with the inhibition of an early step in the formation of autophagosomes. Interestingly, ubiquitination was shown to regulate the activity of the reticulophagy receptor RETREG1/FAM134B by promoting receptor oligomerization [75], which is required for the fragmentation of the ER that enables reticulophagy [76]. Thus, by counteracting both RPL26 UFMylation and the ubiquitination of RETREG1, the vDUB may regulate two critical steps in the reticulophagy pathway. The concomitant activation of ER-stress markers (Figure 8D) is consistent with the notion that reticulophagy plays an important role in alleviating the accumulation of defective translation products in or at the ER [77]. Although attempts to firmly link these effects to the inhibition of UFMylation by showing changes in the abundance of known chaperones and regulators of UFMylation-dependent reticulophagy, such as CDK5RAP3 or CYB5R3, have been so far unsuccessful, possibly due to the low prevalence of ER-stalled ribosomes, these findings provide a notable addition to our earlier observation that BPLF1 inhibits autophagy by regulating the ubiquitination of p62/SQSTM1 [78,79].

While the use of transfection models has been instrumental in our effort to dissect the multiple functions of BPLF1 and has provided new insight into the regulation of RPL26 UFMylation and the ER-RQC, it was important to assess whether the vDUB has similar effects under physiological levels of expression in EBV infected cells. This is a particularly daunting task because BPLF1 is expressed during the early/late phase of the productive virus cycle when multiple viral products contribute to an extensive remodeling of the cell environment. Furthermore, LCL cells are resistant to most transfection procedures, express low levels of many proteins easily detected in the commonly used cell lines, and have a small cytoplasm, which hinders immunofluorescence studies. The severe limitations of the cellular model, together with a presumably low frequency of ER-stalled ribosome stalling during productive infection, explain our failure to conclusively demonstrate the occurrence of endogenous RPL26 UFMylation in induced LCLs. Nevertheless, by comparing a pair of LCLs carrying recombinant EBV encoding catalytic active or inactive BPLF1, we found that physiological levels of the active vDUB inhibited the UFMylation of RPL26 when detectable levels of ribosome stalling were induced by treatment with ANS. The finding is consistent with the notion that the vDUB may regulate the UFMylation-dependent ER-RQC and reticulophagy during productive infection. Inhibition of the ER-RQC could exert a pro-viral function by enhancing the translation of viral membrane proteins and glycoproteins, while the concomitant inhibition of reticulophagy, activation of ER stress responses, and upregulation of ER chaperones could promote protein stabilization and facilitate membrane insertion and proper folding. We have previously reported that, by inhibiting the RQC, BPLF1 enhances the translation of several viral proteins expressed during productive infection [44]. These included the EBV membrane protein LMP1 that traffics through the ER and, due to the presence of multiple transmembrane domains and putative ribosome staling sequences, may trigger the ER-RQC and serve as a reticulophagy substrate. The involvement of this pathway in regulating the expression levels of LMP1 is supported by the finding that, while due to the lack of Lys residues in the coding sequence, LMP1 is a poor proteasomal substrate [80], it is efficiently degraded by autophagy [39]. Collectively, our findings pinpoint a pivotal role of the vDUB in the cellular reprogramming that allows efficient virus production.

## Materials and methods

### Reagents

For a complete list of commercial cell lines, reagents, kits, and commercially available or donated plasmids with source identifiers, see Table 1. The primers used for cloning and qPCR analysis are listed in Table 2.

**Table 1.** Reagents used in this paper.

Reagent	Source	Identifier
Chemicals, peptides, and recombinant proteins		
IGEPAL CA-630	Sigma-Aldrich	I3021; CAS: 9002-93-1
Ciprofloxacin	Sigma-Aldrich	17850; CAS: 85721-33-1
MgCl_2_	Merck	M1028
Sodium dodecyl sulphate	Sigma-Aldrich	L3771; CAS:151-21-3
N-Ethylmaleimide (NEM)	Sigma-Aldrich	E1271; CAS:128-53-0
Iodoacetamide	Sigma-Aldrich	I1149; CAS:144-48-9
Sodium deoxycholate monohydrate	Sigma-Aldrich	D5670; CAS:145224-92-6
Triton X-100	Sigma-Aldrich	T9284; CAS:9002-93-1
Bovine serum albumin	Sigma-Aldrich	A7906; CAS:9048-46-8
Tween-20	Sigma-Aldrich	P9416; CAS: 9005-64-5
PMSF	Sigma-Aldrich	P7626; CAS: 329-98-6
Trizma base	Sigma-Aldrich	93349; CAS:77-86-1
Doxycycline cyclate	Sigma-Aldrich	D9891; CAS: 24390-14-5
IPTG	Saveen Werner AB	A1008; CAS: 367-93-1
Anisomycin (ANS)	Sigma-Aldrich	A5862; CAS:22862-76-6
Cycloheximide	Sigma-Aldrich	C4859; CAS:66-81-9
Carfilzomib	Medchem Express	253339
Bafilomycin A_1_	Sigma-Aldrich	B1793; CAS:88899-55-2
Epoxomicin	Sigma-Aldrich	324801
Thapsigargin	Sigma-Aldrich	T9033; CAS:67526-95-8
Digitonin	Calbiochem	300410
TAK-243	MedChem Express	HY-100487
ML792	MedChem Express	HY-108702
Imidazole	Sigma-Aldrich	I5513; CAS: 288-32-4
Polybrene	Sigma-Aldrich	TR-1003-G;
Complete protease inhibitors cocktail	Roche Diagnostic	04693116001
Phosphatase inhibitor cocktail	Roche Diagnostic	04906837001
DAPI	Sigma-Aldrich	9542; CAS:28718-90-3
Mowiol	Calbiochem	475904; CAS:9002-89-5
Dabco(1,4-Diazabicyclo[2.2.2]octane)	Sigma-Aldrich	D2522; CAS:280-57-9
HA-Ubiquitin-VS	R&D systems	U-212-025
Histone H3.1/H4 Tetramer	NEB Biolabs	M2509S
Glutathione	ThermoFisher Scientific	78259
ATP	ThermoFisher Scientific	R0441
DNase I	Zymo Research	E1009-A
Lysozyme	Thermofisher Scientific	89833
Maltose	MERCK	1.05910 CAS:6363-53-7
**Kits**		
Lipofectamine 2000 transfection kit	Invitrogen	L2000–015
jetOPTIMUS DNA transfection reagent	Polyplus	101000006
Q5® Site-Directed Mutagenesis Kit	New England Biolabs	E0554S
NEBuilder® HiFi DNA Assembly Cloning Kit	New England Biolabs	M5520
Quick-RNA MiniPrep kit	Zymo Research	R1055
RNA clean and concentrator	Zymo Research	R1013
SuperScript VILO cDNA Synthesis kit	Invitrogen	11754050
LC FS DNA Master SYBR Green I	Roche	12239264001
RNaseOUT™ Recombinant Ribonuclease Inhibitor	Thermo Scientific	10777019
DC Protein Assay quantification kit	Bio-Rad	A500–0116
SuperSignal^TM^ WestPico PLUS Chemiluminescent Substrate	Thermo Scientific	XE356732
**Protein purification columns**		
HisPur NiNTA resin	Thermo Scientific	88222
Amylose resin	New England Biolabs	E8021S
Pierce Glutathione Agarose	Thermo Scientific	16101
StrepTrap XT	Cytiva	29401317
Superose 6 hR 10/30	Cytiva	17-0537-01
Microcon-10 Centrifugal filter	Merck	MRCPRT010
**Mediums and Buffers**		
RPMI1640	Sigma-Aldrich	R8758
DMEM	Sigma-Aldrich	D6429
EBSS	Gibco	1854705
HEPES Buffer solution	Gibco	15630–080
**Experimental models: Cell lines**		
HeLa	ATCC	RR-B51S
HEK293T	ATCC	CRL3216
HEK293T *ZNF598* knockout	Liu et al. [44]	N/A
HEK293T *UFSP2* knockout	This paper	N/A
U2OS	ATCC	HTB-96
U2OS *UFSP2* knockout	This paper	N/A
HCT116	ATCC	CCL-247
HCT116-EATR	This paper	N/A
LCL WT/cm (Tet-on BZLF1)	Li et al. [66]	N/A
**Recombinant DNA**		
pCMV3-RPL26-MYC	Sino Biological	HG16834-CM
pCMV3-UFSP2-FLAG	Sino Biological	HG17133-CF
pTRIPZ lentiviral vector	ThermoScientific	RHS4750
pCW57.1	Addgene; gift from David Root	41393
psPAX2	Addgene; gift from Didier Trono	12260
pMD2.G	Addgene; gift from Didier Trono	12259
TetOn-mCherry-eGFP-SERP1	Addgene; gift from Jacob Corn	109014
pLenti-X1-Neo-DDRGK1-WT-HA	Addgene; gift from Jacob Corn	139845
pLenti-X1-Neo-HA-UFL1	Addgene; gift from Jacob Corn	139860
ER-K20	Addgene; gift from Yihong Ye	133861
px330-UFSP2 sgRNA1	Addgene; gift from Yihong Ye	134638
px330-UFSP2 sgRNA2	Addgene; gift from Yihong Ye	134637
pCMV10-3×FLAG-BPLF1	Gastaldello et al. [56]	N/A
pCMV10-3×FLAG-BPLF1^C61A^	Gastaldello et al. [56]	N/A
pMAL-C2X	Addgene; gift from Paul Riggs	75286
pMAL-C2X-UFL1	This paper	N/A
pET28a-His-BPLF1 (1–235)	Gastaldello et al. [85]	N/A
pET28a-His-BPLF1^C61A^	This paper	N/A
pET28a-His-UFSP2	This paper	N/A
pET28a-MBP-UFL1	Ishimura et al. [31]	NA
pACYC-EGFP	Dantuma et al. [58]	N/A
pACYC-Ubiquitin-EGFP	Dantuma et al. [58]	N/A
pACYC-UFM1-EGFP	This paper	N/A
pCDNA3.1-RPL26-S	gift from Ron R. Kopito (Stanford University, Stanford, CA, USA)	N/A
pcDNA3.1-UFM1-GFP	Gift from Yogesh Kulathu (University of Dundee, Dundee, UK)	N/A
pET15b-His-UBA5	Gift from Yogesh Kulathu	N/A
pGX-6P1-GST-UFC1	Gift from Yogesh Kulathu	N/A
pET-15b-His-UFM1	Gift from Yogesh Kulathu	N/A
pETDuet1-His-UFL1-Strep-DDRGK1	Gift from Yogesh Kulathu	N/A

**Table 2.** PCR and qPCR primers.

Gene	Forward primer (5´-3´)	Reverse primer (5´-3´)
pACYC-*UFM1*-EGFP PCR	GTTCCAAAAAGAATTCATCCCCCATGTCGAAGGTTTCCTTT	GGTGCCTCACAAGATCTTCCAACACGATCTCTAGGAATAATCCGC
pMAL-C2X-*UFL1* PCR	CGATCGAGGGAAGGATTTCAGAAATGGCGGACGCCTGGGGAA	CCTGCAGGTCGACTCTAGAGTTACTCTTCCGTCACAGATGATTTCCTAGATTTG
pET28a-His-*UFSP2* PCR	CTGGTGCCGCGCGGCAGCCATATGGTGATTTCAGAAAGTATG	GTCGACGGAGCTCGAATTCGTTAAATCATATTTGGTCGCTG
pET28a-His-*BPLF1*^*C61A*^ PCR	CGGCATCCAGGCAGTCAGCAACTGC	GCAAAGCGGCCAAACTTG
ER_K20 PCR (no FLAG Tag)	GGATCTCCTGGTCCGACCC	TCCGGACTTGTACAGCTCGTC
Spliced *XBP1* qPCR	TGCTGAGTCCGCAGCAGGTG	GCTGGCAGGCTCTGGGGAAG
*HSPA5* qPCR	TGTTCAACCAATTATCAGCAAACTC	TTCTGCTGTATCCTCTTCACCAGT
*GAPDH* qPCR	AAGGTCGGAGTCAACGGATT	CTCCTGGAAGATGGTGATGG

### Antibodies

Mouse monoclonal antibodies: anti-ACTB/β-actin clone AC-15 (1:5000; Sigma-Aldrich, A5441); anti-GAPDH (1:10000; Millipore, CB1001); anti-TUBA/tubulin (1:5000; Millipore, CP06); anti-FLAG (1:10000; Sigma-Aldrich, F3165); anti-S Tag (1:2000; Millipore 71,549–3); anti-HA Tag, (1:2000; Sigma-Aldrich, H9658); anti-RPN1 (1:1000; Santa Cruz Biotechnology, sc -48,367); anti-EIF4G1 Clone 2A9 (1:1000; Abnova, H00001981-M10); anti-ubiquitin, (1:1000; Santa Cruz Biotechnology, sc8017); anti-MYC Tag, clone 4A6, agarose conjugate, (Millipore; 16–219); anti-EBV-BMRF1 (1:10000; Dr. Jaap M. Middeldorp, VU University Medical Center, Amsterdam, NL); Mouse polyclonal antibodies: anti-GFP(B-2) (1:1000; Santa Cruz Biotechnology, sc-9996); anti-FLAG, (1:5000; Sigma-Aldrich, F7425); anti-GFP (1:3000; Abcam, ab290); anti-GFP (1:3000; Abcam, ab290); rabbit monoclonal antibodies: anti-UFC1, (1:5000; Abcam, ab189251); anti-UFM1 (1:3000; Abcam, ab109305); anti-MYC Tag (1:1000; Cell Signaling Technology, 2278); anti-UFM1 (1:3000; Abcam, ab109305); anti-UFC1, (1:5000; Abcam, ab189251); anti-UFSP2 (1:3000; Abcam, ab185965); anti-RPS10 (1:2000; Abcam, ab151550); anti-RPS20 (1:2000; Abcam, ab133776); rabbit polyclonal antibodies anti-ZNF598 (1:1000; Invitrogen, PA559777); anti-LTN1 (1:1000; ProteinTech 28,452–1-AP); anti-UFL1 (1:5000; Bethyl Laboratories, A303-456A); anti-CDK5RAP3 (1:3000; ProteinTech 11,007–1-AP); anti-DDRGK1 (1:3000; Thermo Fisher Scientific, PA5–100467); anti-histone H3 (1:2000; Millipore, 07–690); anti-SEC63 (1:2000; Bethyl Laboratories, A305-084A); anti-SRPRB (1:2000; Bethyl Laboratories, A305-440A); anti-SRP68 (1:2000; Bethyl Laboratories, A303-955A); anti-RPL26, (1:2000; Abcam, ab59567); anti-SEC61B (1:10000; Thermo Fisher Scientific, PA3–015); anti-CYB5R3 (1:2000; ProteinTech 10,894–1-AP); anti-LC3B (1:1000; Sigma-Aldrich, L7543); anti-SUMO2 + 3 (1:1000; Abcam, ab3742); anti-FLAG M2 affinity gel (Sigma-Aldrich, A2220); S-Protein Agarose, (Novagen 69,704); GammaBind Plus Sepharose (Cytiva 17,088,601); Rabbit IgG isotype control (Abcam, ab172730); Goat Anti-Rabbit IgG (H+L) Antibody, Alexa Fluor 647 Conjugated (1:1000; Thermo Fisher Scientific, A21245).

### Tandem mass spectrometry and bioinformatics analysis

The mass spectrometry characterization of the BPLF1 interactome was reported previously [44]. For the current analysis, proteins detected by at least one unique spectral count in both the FLAG-BPLF1 or -BPLF1^C61A^ immunoprecipitates that were either not detected in immunoprecipitates of FLAG-ev transfected cells or showed Log_2_ fold increase ≥ 1.5 were considered as positive hits. The bioinformatics resource Search Tool for the Retrieval of Interacting Genes (STRING v9.0) and Database for Annotation, Visualization, and Integrated Discovery (DAVID v6.8) were used to identify the representation of genes in particular functional categories. Analysis of biological process (BP) terms was performed using the ToppCluster [81] and Gene Ontology [82,83] databases. Protein interaction network analysis was performed using the STRING database, followed by manual curation. The interaction network was visualized using Cytoscape v3.10.1.

### Plasmid construction

Previously described, purchased, and donated plasmids with identifiers and references are listed in Table S2. The ER-K20 reporter was modified by removing the FLAG-Tag using the Q5® Site-Directed Mutagenesis Kit according to the recommended protocol. A plasmid for prokaryotic expression of UFSP2 was constructed by extracting the UFSP2 coding sequence from the pCMV3-UFSP2-FLAG plasmid by PCR, followed by in-frame insertion into the pET28a+ vector (Merck 70,777) using the NEbuilder HiFi DNA Assembly Master Mix. A plasmid for procaryotic expression of the catalytic mutant BPLF1^C61A^ was produced by site-directed mutagenesis of the pET28a-His-BPLF1 plasmid. A UFM1-GFP plasmid for bacterial expression was produced by extracting the UFM1ΔC2 coding sequence from pcDNA3-MYC-UFM1ΔC2 by PCR. The PCR product was digested with EcoRI and BglII and inserted in-frame into the recipient vector pACYCduet-eGFP plasmid [58]

### Cell lines

The HeLa, HEK293T, U2OS, and HCT116 cell lines purchased from ATCC were cultured in Dulbecco’s minimal essential medium (DMEM) supplemented with 10% FBS and 10 μg/ml ciprofloxacin (complete medium) and grown in a 37°C incubator with 5% CO_2_. The TetOn-BZLF1-EBV immortalized LCLs expressing catalytically active or inactive BPLF1, and a tetracycline-regulated BZLF1 transactivator [66] were cultured in RPMI1640 medium supplemented with 10% Tet-free FBS. The productive virus cycle was induced by treatment with 1.5 µg/ml doxycycline for 72 h, and induction efficiency was routinely verified by probing western blots with antibodies to the early antigen BMRF1. The cell lines were transfected using either the Lipofectamine 2000 (Invitrogen, L2000–015) or jetOPTIMUS® DNA transfection reagents according to the protocols recommended by the manufacturers. A stable HCT116 subline expressing the TetOn-mCherry-eGFP-SERP1 reticulophagy tandem fluorescence reporter (HCT116-EATR) was produced by lentivirus transduction. Briefly, the TetOn-mCherry-eGFP-SERP1, psPAX2, and pMD2.G plasmids were cotransfected into HEK293T cells using the JetOPTIMUS® kit. After culture overnight in Tet-free complete medium, the medium was refreshed, and the cells were cultured for an additional 48 h to allow virus production. The virus-containing culture supernatant was briefly centrifuged and passed through a 0.45-µm filter to remove cell debris before aliquoting and storing at −80°C until use. HCT116 were infected with virus-containing supernatants for 24 h in the presence of 8 μg/ml polybrene, followed by replacing the infection medium with a fresh complete medium. Transduced cells were sorted twice by fluorescence-activated cell sorting (FACS) based on ChFP and eGFP fluorescence, and stably expressing cells were cultured in Tet-free DMEM complete medium. HEK293T- and U2OS-*UFSP2*-knockout cells were generated by co-transfection with the px330-UFSP2 sgRNA1 and px330-UFSP2 sgRNA2 plasmids and a GFP-expressing plasmid. After two rounds of FACS sorting based on GFP expression, *UFSP2* knockout was confirmed by probing immunoblots with a UFSP2-specific antibody. The establishment of the HEK293T-*ZNF598*-knockout cell line was described previously [44]. HEK293-*LTN1*-knockdown cells were produced by siRNA transfection. Briefly, HEK293T cells were seeded into a 6-well plate and transfected with 20 nM *LTN1*-specific siRNA (siRNA ID: s25003; Invitrogen 4,392,420) by Lipofectamine 2000 according to the manufacturer’s protocol. As a control, the cells were transfected with Silencer™ Select Negative Control No. 2 siRNA (Invitrogen 4,390,847). The medium was refreshed after 24 h, and the cells were cultured for 48 h. The knockdown efficiency was validated by immunoblot with an LTN1-specific antibody.

### Immunoblotting, co-immunoprecipitation, and affinity-isolation assays

Cells were incubated for 30 min on ice in NP-40 lysis buffer (50 mM Tris-HCl, pH 7.6, 150 mM NaCl, 5 mM MgCl_2_, 1 mM EDTA, 1% Igepal CA-630, 5% glycerol) supplemented with protease inhibitor cocktail. To detect ubiquitination or UFMylation, the activity of cellular deconjugases was inhibited by supplementing the lysis buffer with 20 mM NEM and 20 mM iodoacetamide. After centrifugation at 20,000 × g for 15 min at 4°C, the protein concentration of the supernatants was measured with a protein assay kit. Equal amounts of proteins were fractionated in acrylamide Bis-Tris 4–12% gradient gel (Invitrogen, NP0321). After transfer to PVDF membranes, the blots were blocked in Tris-buffered saline (TBS; Santa Cruz Biotechnology, C2624) containing 0.1% Tween-20 and 5% nonfat milk. The membranes were incubated with the primary antibodies diluted in blocking buffer for 1 h at room temperature or overnight at 4°C, followed by washing and incubation for 1 h with the appropriate horseradish peroxidase-conjugated secondary antibodies. The immunocomplexes were visualized by enhanced chemiluminescence. For immunoprecipitation, the cells were harvested 24 h after transfection and lysed in IP lysis buffer (20 mM Tris-HCl, pH 7.6, 150 mM NaCl, 1 mM EDTA, 1% Igepal CA-630, 1% Triton X-100) supplemented with protease inhibitor cocktail for 30 min on ice. For immunoprecipitations under denaturing conditions, the cells were lysed for 10 min on ice in one-tenth of the final volume of lysis buffer supplemented with 1% SDS, followed by dilution to 0.1% SDS. For co-immunoprecipitation, the NP-40 lysates were incubated with 50 μl) anti-FLAG or anti-MYC conjugates agarose affinity gel for 3 h at 4°C with rotation. For co-immunoprecipitation of endogenous UFL1, DDRGK1, and CDK5RAP3, the NP-40 lysates were incubated for 4 h with the specific antibodies, followed by capture of the immunocomplexes with protein-G coupled Sepharose beads. The beads were washed with lysis buffer, and the immunocomplexes were eluted by boiling in 2×NuPAGE Loading buffer supplemented with a sample-reducing agent. All images were acquired using a ChemiDoc Imaging system (Bio-Rad), and the intensity of target bands was quantified using the ImageLab software. For in vitro affinity-isolation assays, UFL1 was extracted from the pLenti-X1-Neo-HA-UFL1 plasmid by PCR and cloned into the pMAL-C2X plasmid using the hi-fi assembly system (New England Biolabs, M5520). Recombinant MBP-UFL1 and His-BPLF1 [56] were expressed in transformed BL21 cells cultured with 10 mM IPTG. The bacterial cells were then resuspended in buffer A (25 mM Tris, pH 8.0, 300 mM NaCl, 10% glycerol, 5 mM DTT, 50 IU DNAse, 0.1 mg/mL lysozyme, protease-inhibitor cocktail) and lysed by sonication with 5 cycles of 30 s on 20 s off pulses at 20 kHz on ice. The lysates were cleared by centrifugation at 20,000 × g for 20 min at 4°C and followed by incubation with NiNTA-agarose beads or amylose resin (NEB biolabs, E8021S) at 4°C for 1 h. The MBP-UFL1, mixture was loaded on a gravity column and washed with 10 mL of buffer A followed by elution with a gradient of maltose in buffer A (0.1–10 mM maltose). The proteins were purified by size-exclusion chromatography on a Superose 6 hR 10/30 column in base buffer, concentrated on Microcon-10 filters, and stored in 25 mM Tris, 50 mM NaCl, 10% glycerol, 2 mM DTT, pH 7.5 at −80°C until use.

### In vitro UFMylation assays

The recombinant His-BPLF1, His-UFSP2, His-UBA5, GST-UFC1, His-UFM1, and His-UFL1/Strep-DDRGK1 proteins and in vitro UFMylation reactions were produced as described [30]. Briefly, the proteins were expressed in BL21 bacterial cells by overnight incubation at 16°C with 10 μM IPTG. The bacterial cells were lysed in Base buffer (25 mM Tris, 300 mM NaCl, 10% glycerol, 2 mM DTT, pH 8.0) supplemented with a complete protease-inhibitor cocktail by sonication with 5 cycles of 30 s on 20 s off pulses at 20 kHz on ice. The lysates were centrifuged at 20,000 × g for 20 min at 4°C. Proteins were purified using NiNTA- or Glutathione-agarose beads. Imidazole and glutathione were removed by dialysis against the base buffer. The proteins were purified by size-exclusion chromatography on a Superose 6 hR 10/30 column on the base buffer, concentrated on Microcon-10 filters, and stored in 25 mM Tris, 50 mM NaCl, 10% glycerol, 2 mM DTT, pH 7.5 at −80°C until use. For the UFMylation reactions, 0.25 μM His-UBA5, 5 μM GST-UFC1, 1 μM His-UFL1/Strep-DDRGK1, 0.5 μM of H3/H4 tetramer and 10 μM His-UFM1 were incubated in reaction buffer (50 mM HEPES, 10 mM MgCl_2_, 5 mM ATP, pH 7.4), in the absence or presence of BPLF1 or UFSP2, at 37°C for 90 min. The reactions were stopped by adding LDS-sample buffer supplemented with 100 mM DTT, and immunoblots were probed with the indicated antibodies.

### Deufmylase assay

U2OS-*UFSP2*-knockout cells were cotransfected with a reporter plasmid expressing a UFM1-GFP fusion protein and plasmids expressing FLAG-BPLF1 or FLAG-UFSP2. After overnight culture, the cells were lysed on RIPA buffer (50 mM Tris, 150 mM NaCl, 1% Igepal, 0.5% deoxycholic acid, 0.1% SDS, pH 7.4) supplemented with complete protease inhibitors cocktail and 20 mM NEM. The lysates were cleared by centrifugation at 21,130 × g for 30 min at 4°C, fractionated by SDS-PAGE, and transferred to PVDF membranes. Proteins were detected by immunoblotting with specific antibodies.

### Cell fractionation by sequential detergent extraction

Cells were resuspended in 150 µL fractionation buffer (50 mM HEPES, pH 7.35, 150 mM NaCl with protease inhibitor cocktail and 1 mM PMSF (Sigma-Aldrich, P7626). One-third of the sample was lysed in fractionation buffer containing 1% Igepal CA-630 for 10 min on ice, followed by centrifugation for 10 min at 21,130 × g at 4°C to obtain a clear whole-cell lysate sample (W). The remaining two-thirds of the sample was resuspended in 100 µL fractionation buffer containing 0.005% digitonin for 10 min to permeabilize the plasma membrane, followed by centrifugation at 8000 × g for 5 min. The supernatant containing the cytosolic fraction (C) was collected, and the pellet was washed once with 500 µL fractionation buffer with no detergents before resuspending in 100 µL fractionation buffer containing 1% Igepal CA-630. After incubating for 10 min on ice and centrifugation at 21,130 × g for 10 min, the supernatant containing the membrane fraction (E) was collected.

### Polysome profiling

HEK293T grown to ~ 80% confluence on 10-cm tissue culture plates were transfected with FLAG-ev or -BPLF1 for 24 h and then treated with ANS as described above. Polysome-fractionation was performed as described [84]. Briefly, cells were rinsed with ice-cold 1× PBS containing 100 µg/mL cycloheximide (CHX), harvested by scrapping in PBS/CHX and pellet by centrifugation at 300 × g for 5 min at 4°C. The cells were then lysed with hypotonic lysis buffer (5 mM Tris pH 7.5, 2.5 mM MgCl_2_, 1.5 mM KCl, 2 mM dithiothreitol, DTT, 0.5% Triton X-100, 0.5% sodium deoxycholate, 100 μg/ml CHX, and 120 U RNaseOUT). After centrifugation at 16,025 × g for 2 min at 4°C, cleared lysates were layered onto a linear 5–50% sucrose density gradient (100 mM HEPES, pH 7.6, 500 mM KCl, 25 mM MgCl_2_) prepared using a BioComp gradient master. Gradients were centrifuged in a Beckman SW41 rotor at 22,000 × g for 2 h at 4°C, fractionated, and 500 µl fractions were collected while monitoring the absorbance at 254 nm using a Gilson FC 203B Fraction Collector. One mL of absolute ethanol was added to the collected fractions that were then kept at −20°C overnight. The samples were centrifuged at 8176 × g for 15 min at 4°C and then washed with 70% ethanol. The pellets were resuspended in Laemmli buffer and boiled for 10 min before SDS-PAGE and immunoblot analysis.

### ER-RQC reporter assay and Endo H treatment

The ER-K20 reporter was cotransfected in HEK293T cells with either FLAG-ev, -BPLF1 or -BPLF1^C61A^. After 24 h, the cells were lysed in RIPA lysis buffer (50 mM Tris, 150 mM NaCl, 1% Igepal, 0.5% deoxycholic acid, 0.1% SDS, pH 7.4), and protein expression was assessed by immunoblot. Where indicated, 200 nM bafilomycin A_1_, 100 nM epoxomicin, or 200 nM carfilzomib were added to the cultures overnight to inhibit lysosome or proteasome-dependent degradation, respectively. To assess peptide glycosylation, aliquots of the cell lysates were treated with endoglycosidase H (Endo H; New England Biolabs, P0702S) according to the manufacturer’s recommendation. Briefly, 10 μg of total cell lysates were denatured at 100°C for 10 min with glycoprotein denaturing buffer. The denatured lysates were treated with 500 units of Endo H at 37°C for 1 h. The reactions were stopped by adding NuPAGE loading buffer plus reducing agent and boiling. Samples were analyzed using NuPAGE 4–12% gradient gel.

### ER Autophagy Tandem Reporter (EATR) assay

The expression of the EATR reporter was induced in HCT116-EATR cells grown on cover slides by treatment for 24 h with 2 μg/ml doxycycline before FLAG-BPLF1 or -BPLF1^C61A^ transfection. After culture for an additional 24 h, the cells culture medium was removed by repeated PBS (Sigma-Aldrich, D8573) washing, and the cells were starved by culture for 16 h in Earl’s balanced salt solution (EBSS) medium in the presence or absence of 200 nM bafilomycin A_1_. The cells were then fixed and stained with the anti-FLAG antibody as described. Images were acquired using a Zeiss LSM900 confocal microscope, and the number of red or yellow dots in 45 BPLF1 or BPLF1^C61A^-positive or -negative cells was counted manually.

### Quantitative polymerase chain reaction (qPCR)

Total RNA was extracted with the Quick-RNA MiniPrep kit within in-column DNase treatment according to the manufacturer’s instructions. One μg total RNA was reverse transcribed using the SuperScript VILO cDNA Synthesis kit. The relative mRNA level of target genes was measured on a LightCycler 1.2 instrument (Roche Diagnostic) using the LC FastStart DNA master SYBR Green I kit with specific primers (Table S2). All reactions were performed in duplicate. The mRNA level of the housekeeping gene *GAPDH* was used as an internal control. The relative fold change of gene expression was determined with the comparative cycle threshold (2^−ΔΔCT^) method.

### Statistical analysis

Plotting and statistical tests were conducted with data obtained in two or more independent experiments using the Microsoft Excel or GraphPad Prism softwares. No assumptions about data normality were made, and unpaired two-tailed Student’s t-tests were used to determine statistical significance.

## Supplementary Material

Liu et al Supplementary Information R3.docx

## Data Availability

All data generated and analyzed during this study are included in this published article and its Supplementary files. Source data are included in the Source Data file. Processed mass spectrometry data and details of the databases, softwares, and analysis procedure are provided in Supplementary Data 1, Liu et al. Nature Communications volume 14, Article number: 8315 (2023).
