# Supplementary material for: The Epstein-Barr virus deubiquitinase BPLF1 regulates stress-induced ribosome UFMylation and reticulophagy
Source: Autophagy. 2025 Jan 22;21(5):996–1018. doi: 10.1080/15548627.2024.2440846 (PMC12013442; doi:10.1080/15548627.2024.2440846)
Supplement: Liu et al Supplementary Information R3.docx [file KAUP_A_2440846_SM9294.docx]

**SUPPLEMENTARY INFORMATION**

| **Table S1.** BPLF1-interacting proteins identified by tandem mass spectrometry. | | | | |
| --- | --- | --- | --- | --- |
| **UniProt ID** | **Symbol** | **Protein name** | **Log_2_ fold change** | **Average peptides** |
| Q04637 | **EIF4G1** | eukaryotic translation initiation factor 4 gamma 1 | 22.5 | 6.5 |
| Q99613 | **EIF3C** | eukaryotic translation initiation factor 3 subunit C | 11.4 | 5.5 |
| Q9UGP8 | **SEC63** | SEC63 homolog, protein translocation regulator | 9.3 | 2.5 |
| P62280 | **RPS11** | ribosomal protein S11 | 6.5 | 2 |
| Q9Y5M8 | **SRPRB** | SRP receptor subunit beta | 5.6 | 6.5 |
| P55072 | **VCP** | valosin containing protein | 5.2 | 3 |
| P78344 | **EIF4G2** | eukaryotic translation initiation factor 4 gamma 2 | 4,4 | 2 |
| Q9UBQ5 | **EIF3K** | eukaryotic translation initiation factor 3 subunit K | 3.9 | 1 |
| Q15056 | **EIF4H** | eukaryotic translation initiation factor 4H | 3.8 | 1.5 |
| P55010 | **EIF5** | eukaryotic translation initiation factor 5 | 3.7 | 1 |
| P04843 | **RPN1** | ribophorin I; protein glycosyltransferase subunit 1 | 3.5 | 7 |
| Q9UI10 | **EIF2B4** | eukaryotic translation initiation factor 2B subunit delta | 3.2 | 1.5 |
| Q9BY44 | **EIF2A** | eukaryotic translation initiation factor 2A | 3.1 | 1.5 |
| O94874 | **UFL1** | UFM1 specific ligase 1; E3 | 3 | 2.5 |
| Q8N766 | **EMC1** | ER membrane protein complex subunit 1 | 2.8 | 2 |
| P62899 | **RPL31** | ribosomal protein L31 | 2.7 | 1.5 |
| P29692 | **EEF1D** | eukaryotic translation elongation factor 1 delta | 2.6 | 1 |
| P05387 | **RPLP2** | ribosomal protein lateral stalk subunit P2 | 2.5 | 2 |
| Q9H9T3 | **ELP3** | elongator acetyltransferase complex subunit 3 | 2.4 | 1 |
| P05198 | **EIF2A** | eukaryotic translation initiation factor 2A | 2.3 | 3 |
| Q14152 | **EIF3A** | eukaryotic translation initiation factor 3 subunit A | 2.3 | 10 |
| P84098 | **RPL19** | ribosomal protein L19 | 1.9 | 2 |
| P61254 | **RPL26** | ribosomal protein L26 | 1.9 | 3 |
| P08708 | **RPS17** | ribosomal protein S17 | 1.8 | 1 |
| P46782 | **RPS5** | ribosomal protein S5 | 1,8 | 2 |
| Q15436 | **SEC23A** | SEC23 homolog A, COPII coat complex component | 1.8 | 1 |
| P37108 | **SRP14** | signal recognition particle 14 | 1.8 | 1 |
| Q969S9 | **GFM2** | GTP dependent ribosome recycling factor mitochondrial 2 | 1.7 | 1 |
| Q9NRP0 | **OSTC** | oligosaccharyltransferase complex non-catalytic subunit | 1.6 | 1 |
| Q9Y3U8 | **RPL36** | ribosomal protein L36 | 1.6 | 2 |
| O76094 | **SRP72** | signal recognition particle 72 | 1.6 | 1 |
| Q86UK7 | **ZNF598** | zinc finger protein 598, E3 ubiquitin ligase | 1.6 | 1 |
| P62847 | **RPS24** | ribosomal protein S24 | 1.5 | 1.5 |
| P55735 | **SEC13** | SEC13 homolog, nuclear pore and COPII coat complex component | 1.5 | 1 |

**SUPPLEMENTARY FIGURES**

**Figure S1.** Time and amount of ANS required for induction of RPL26 UFMylation. (**A**) The cell lines were treated with the indicated ANS concentrations for 20 min before analysis of RPL26 UFMylation (identified based on the size of the bands detected by the UFM1 antibody) and RPS10 and RPS20 ubiquitination. Immunoblots from one representative experiment out of two are shown in the figure. (**B**) HEK293T cells transfected with FLAG-ev, -BPLF1 or -BPLF1^C61A^ were treated with 50 ng/ml ANS before western blot analysis. Maximal levels of UFMylation were observed after treatment for 20 min, and the levels remained constant over time. Immunoblots from one representative experiment out of two are shown.

**50**

**37**


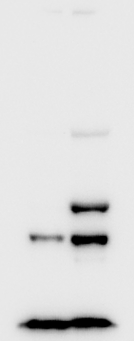

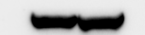

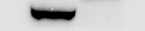


**UFSP2**

**ACTB**

**ctr**

***UFSP2*-KO**

**kDa**

**UFM1**

**250**

**150**

**100**

**75**

**50**

**37**

**25**

**20**

**15**

**10**

**A**

**50**

**37**

**25**

**20**

**25**

**37**

**50**


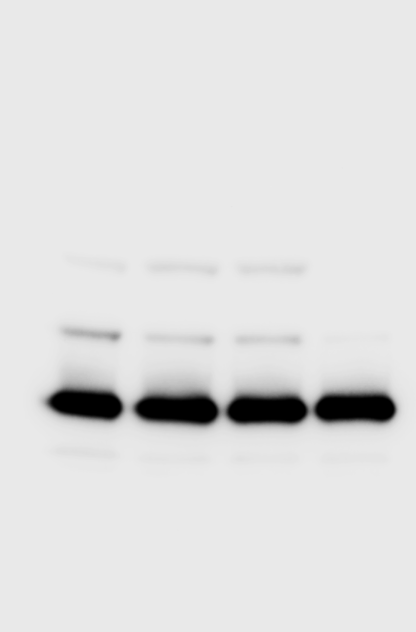


**RPL26**

**+**

**-**

**-**

**-**

**-**

**+**

**-**

**-**

**-**

**-**

**+**

**-**

**-**

**-**

**-**

**+**

**kDa**

**FLAG-ev**

**FLAG-BPLF1**

**FLAG-BPLF1^C61A^**

**FLAG-UFSP2**

**UFM1-RPL26**

**GAPDH**

**FLAG**

**UFSP2**


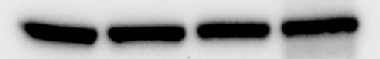

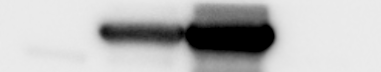

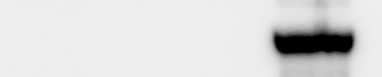


**B**

**Figure S2.** Characterization of the U2OS-*UFSP2*-KO cell lin**e**. (**A**) The main cellular de-UFMylase UFSP2 was knocked out in U2OS cells by co-transfection with GFP and px330-*UFSP2*-sgRNAs expressing plasmids. After two rounds of FACS sorting, the expression of UFSP2 was assessed in immunoblots. A representative immunoblot that illustrates the loss of UFSP2 and concomitant accumulation of endogenous UFMylated substrates is shown. (**B**) BPLF1 does not promote the deUFMylation of endogenous RPL26 in *UFSP2*-KO cells. The *UFSP2*-KO cells were cotransfected with FLAG-ev, -BPLF1 or -BPLF1^C61A^ or FLAG-UFSP2, and immunoblots were probed with the RPL26 antibody. Immunoblots from one representative experiment out of two are shown.


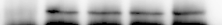

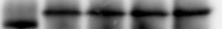

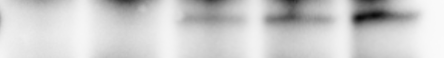

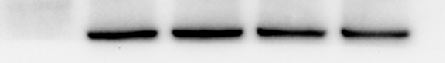


**FLAG-BPLF1 (μg)**

**0**

**2**

**4**

**8**

**input**

**0**

**2**

**4**

**8**

**IP DDRGK1**

**IgG**


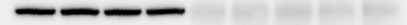


**ACTB**


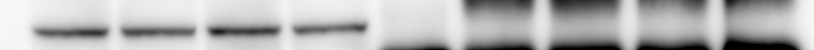


**CDK5RAP3**


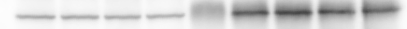


**DDRGK1**


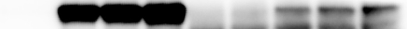


**FLAG**


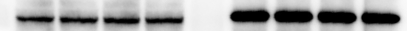


**UFL1**


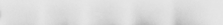


**0**

**2**

**4**

**8**

**IgG**

**IP UFL1**

**25**

**37**

**37**

**100**

**50**

**kDa**

**Figure S3.** Overexpressed BPLF1 does not affect the assembly of the UFM1 ligase complex. HEK293T cells were transfected with increasing amounts of the FLAG-BPLF1 plasmid. Cell lysates were immunoprecipitated with the indicated antibodies. Immunoblots from one out of two independent experiments are shown in the figure.

**Figure S4.** Validation of the ubiquitin and SUMO E1 inhibitors. HEK293T cells were treated with the indicated concentrations of TAK243 or ML-792 for 3 h before treatment with 50 ng/ml ANS for 20 min. Immunoblots of cell lysates from the experiment shown in Figure 6C were probed with the indicated antibodies.

**Figure S5.** BPLF1 cofractionates with ribosomes and is enriched on polysomes. (**A**) Sucrose gradient sedimentation of untreated HEK293T cells and cells treated with ANS in the absence or presence of catalytically active BPLF1. Polysome tracing from one out of two independent experiments is shown. The peak fractions enriched in 40S, 60S, 80S ribosomes, and first polysome fraction are indicated by arrows. (**B**) Collected fractions were analyzed by immunoblotting. The 40S and 60S ribosome proteins were identified by probing the blots with RPS20- and RPL26-specific antibodies. Although, as expected, a large proportion of the transfected BPLF1 was recovered in the pre-ribosomal fractions, the vDUB was detected in all ribosome-containing fractions and enriched in the polysome fractions.


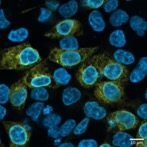

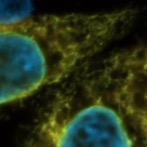

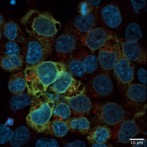

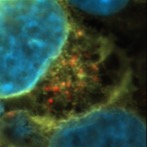


**Dox**

**Dox^+^ starved**

**Blow-up 5x**

**ChFP-GFP**

**B**


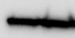


**GAPDH**

**GFP/**

**ChFP**


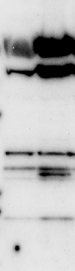


**75**

**50**

**37**

**25**

**20**

**15**

**10**

**Dox - +**

**A**

**KDa**


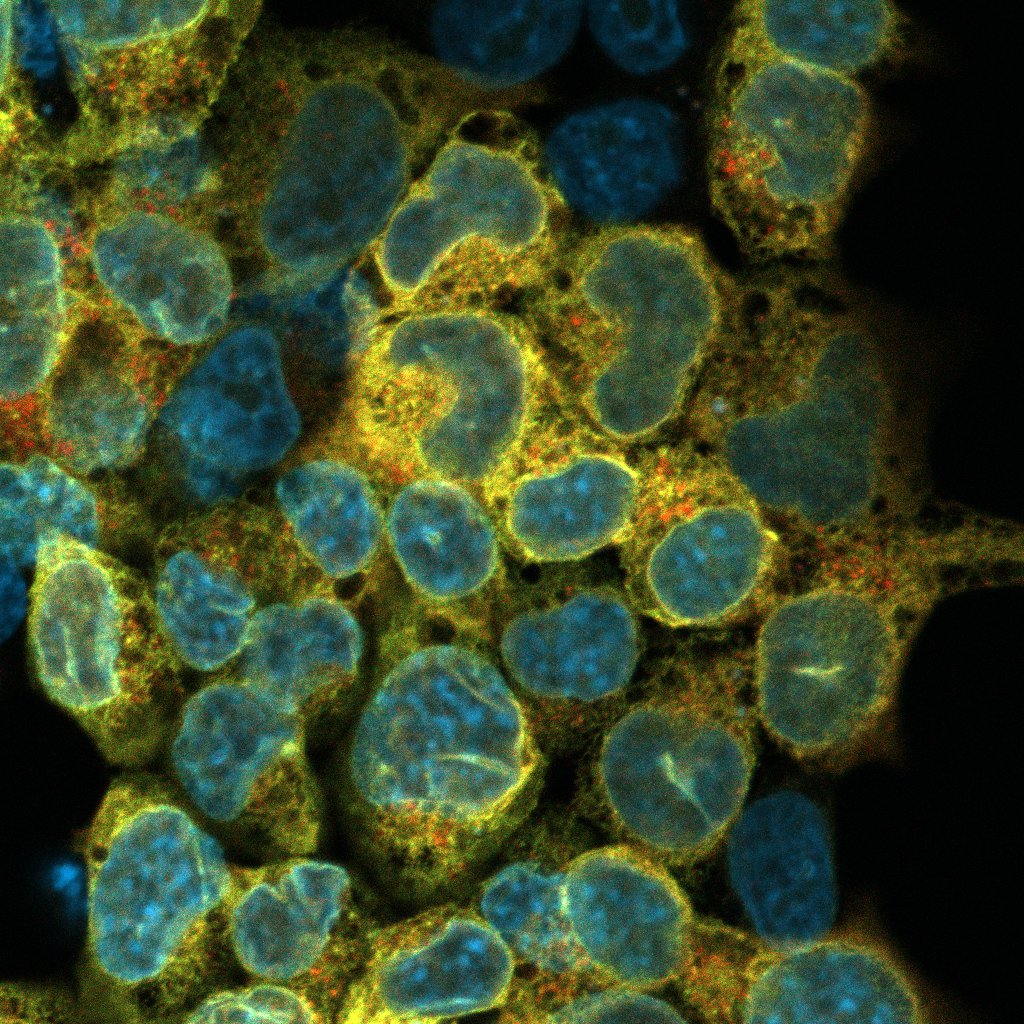

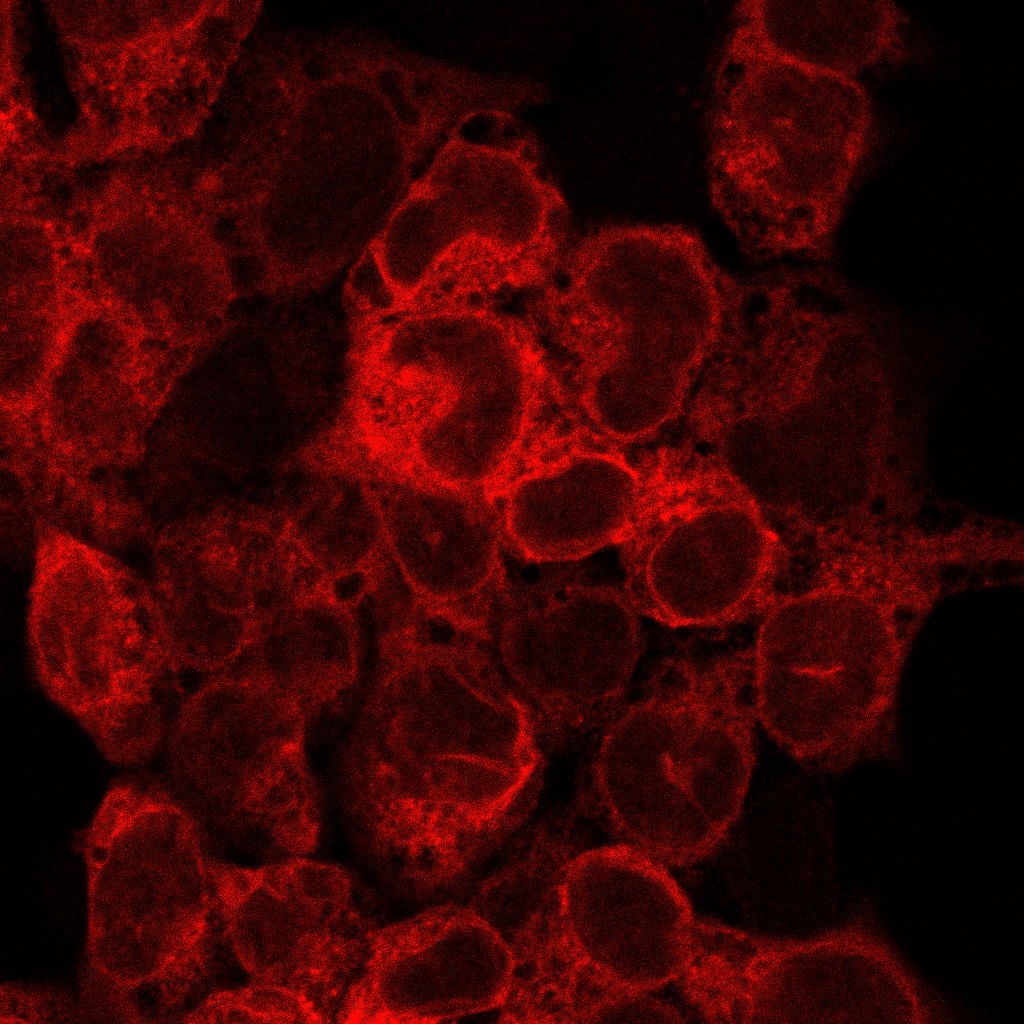

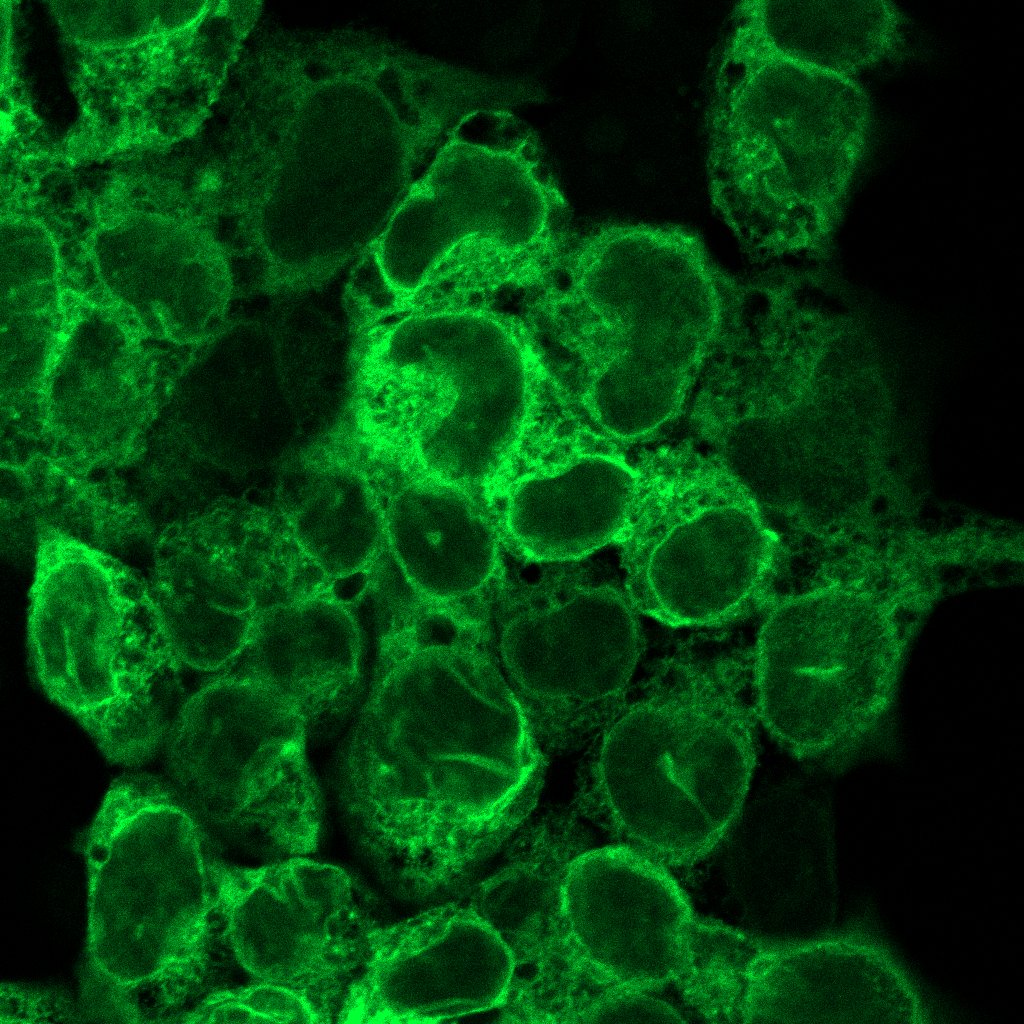

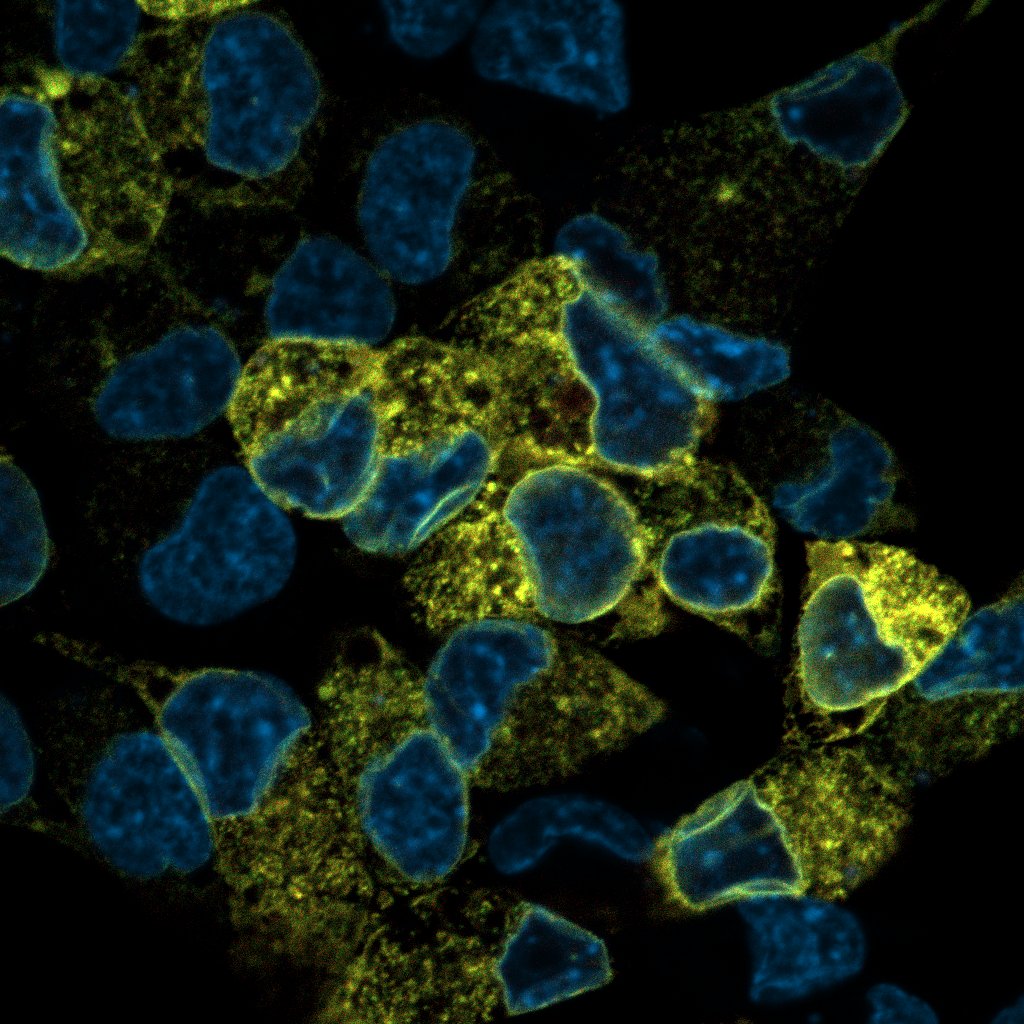

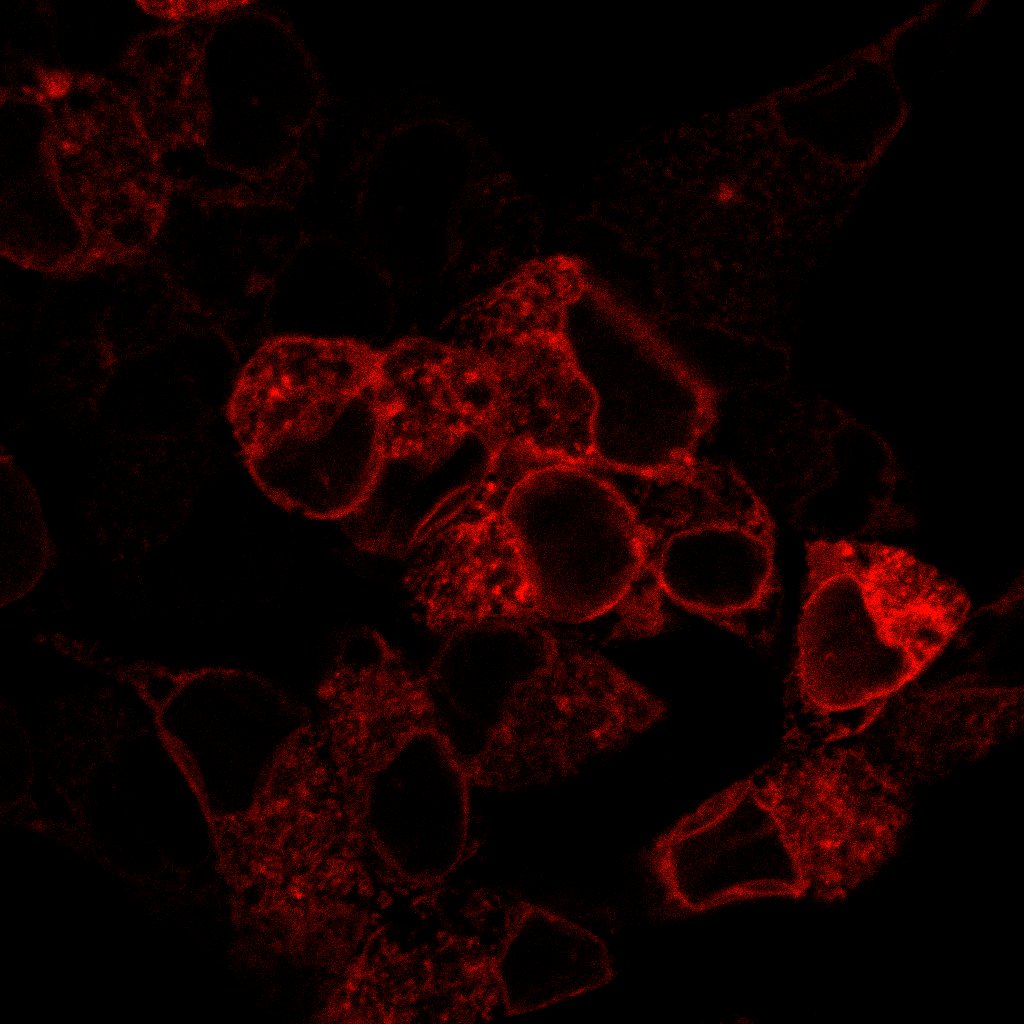

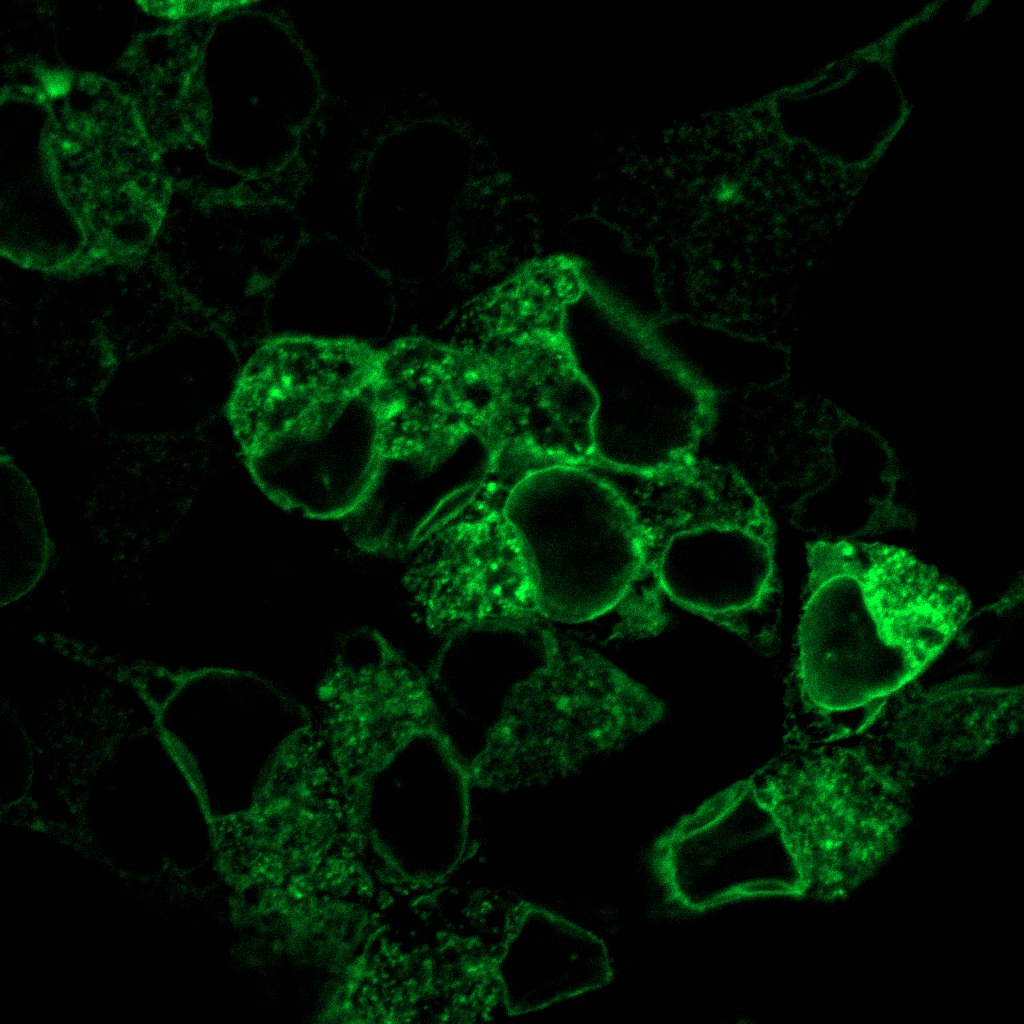

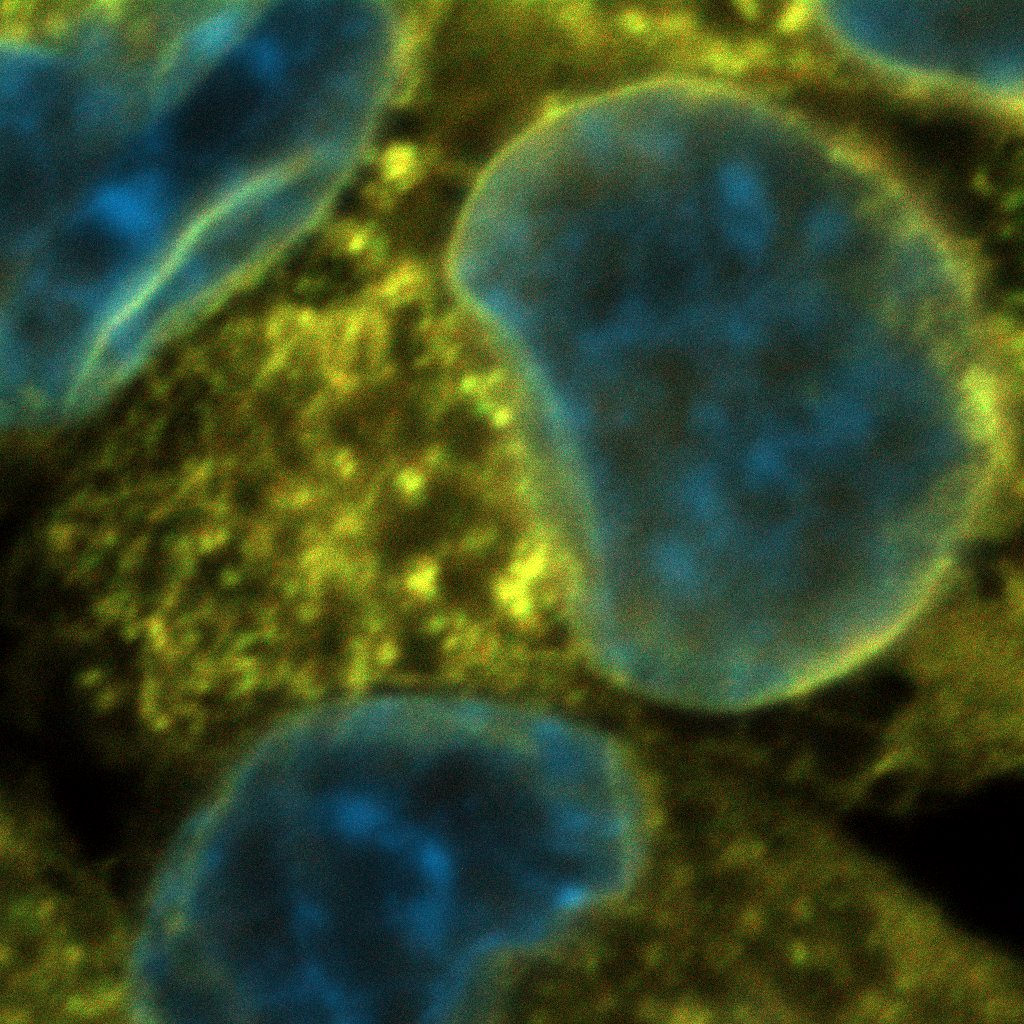

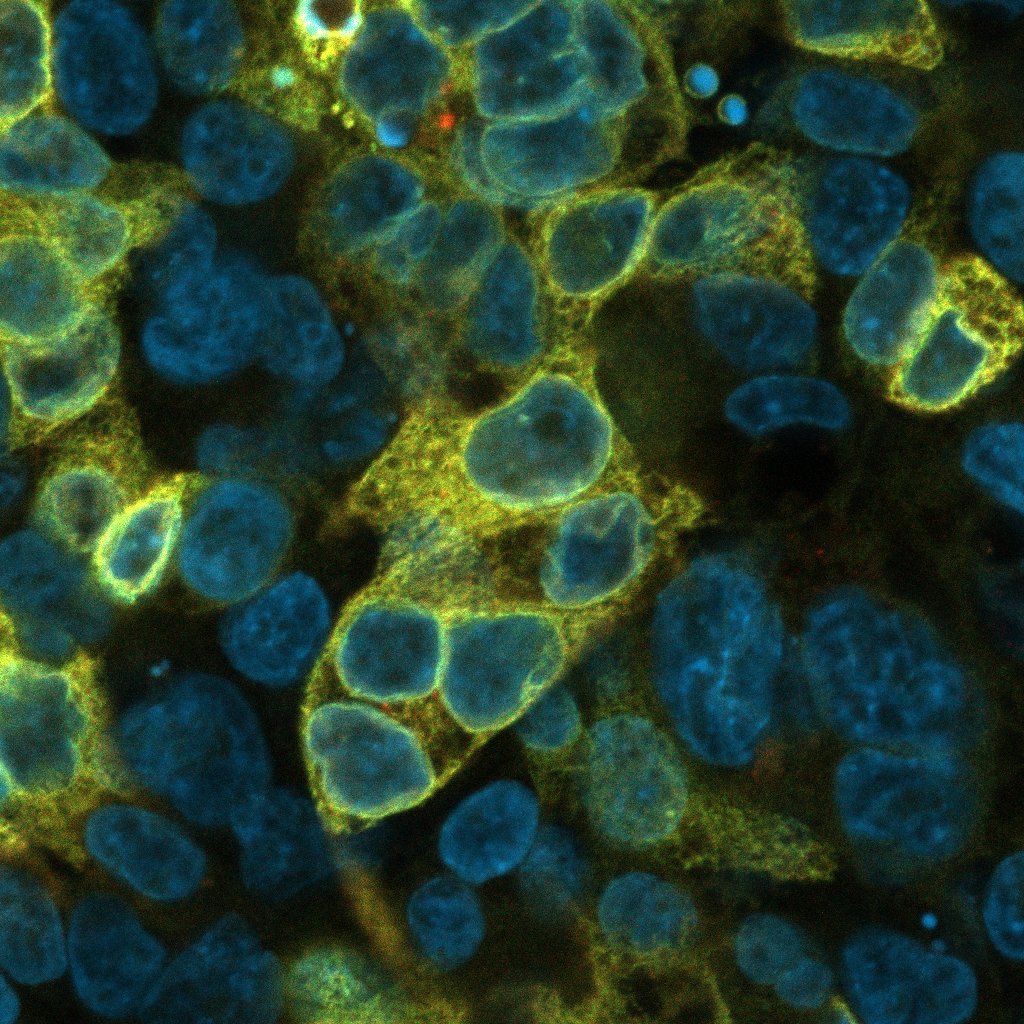

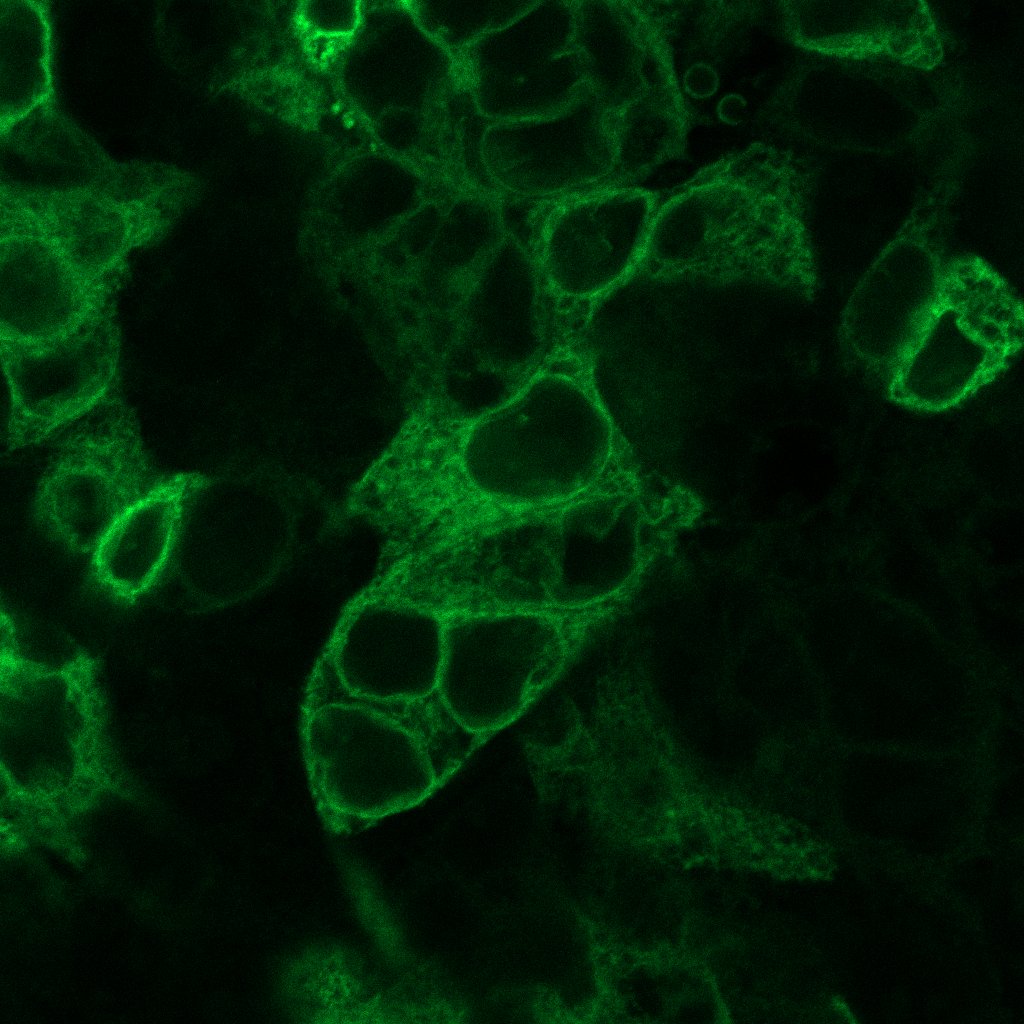

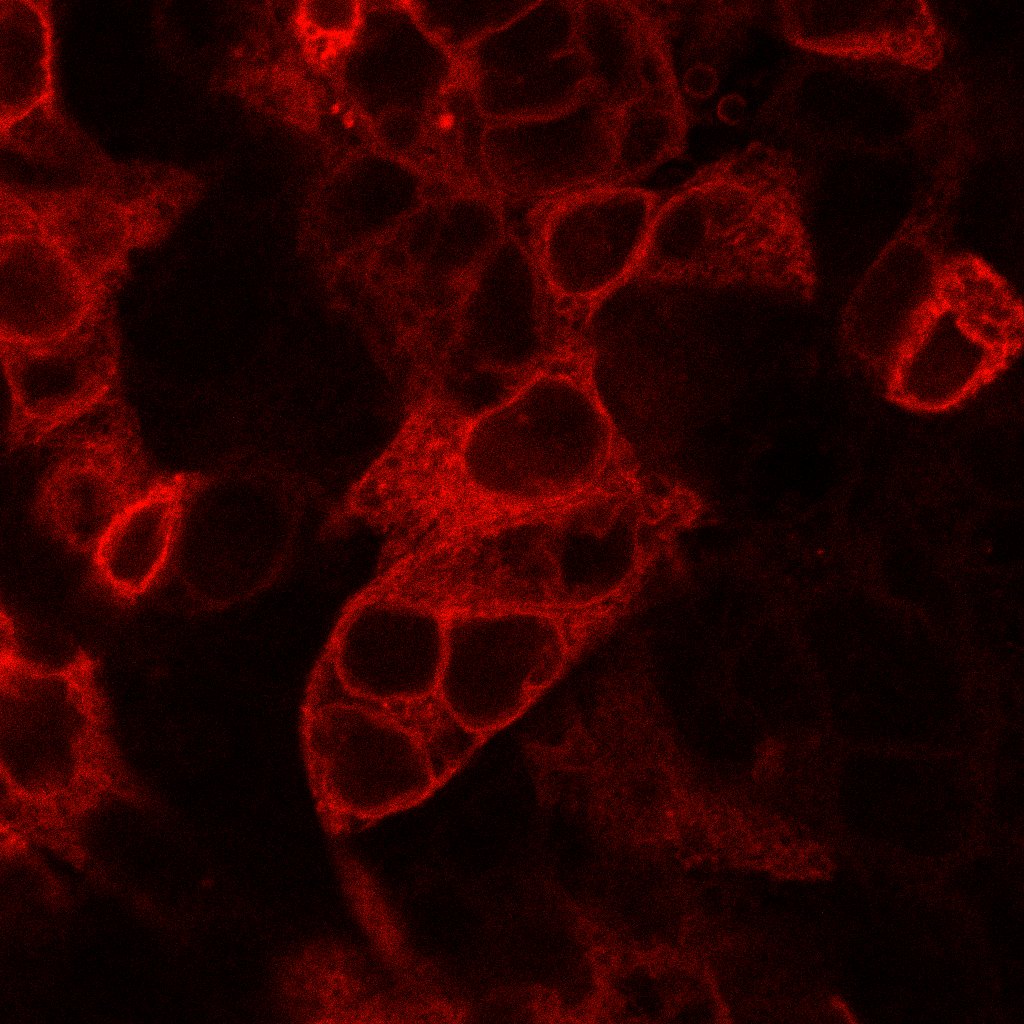

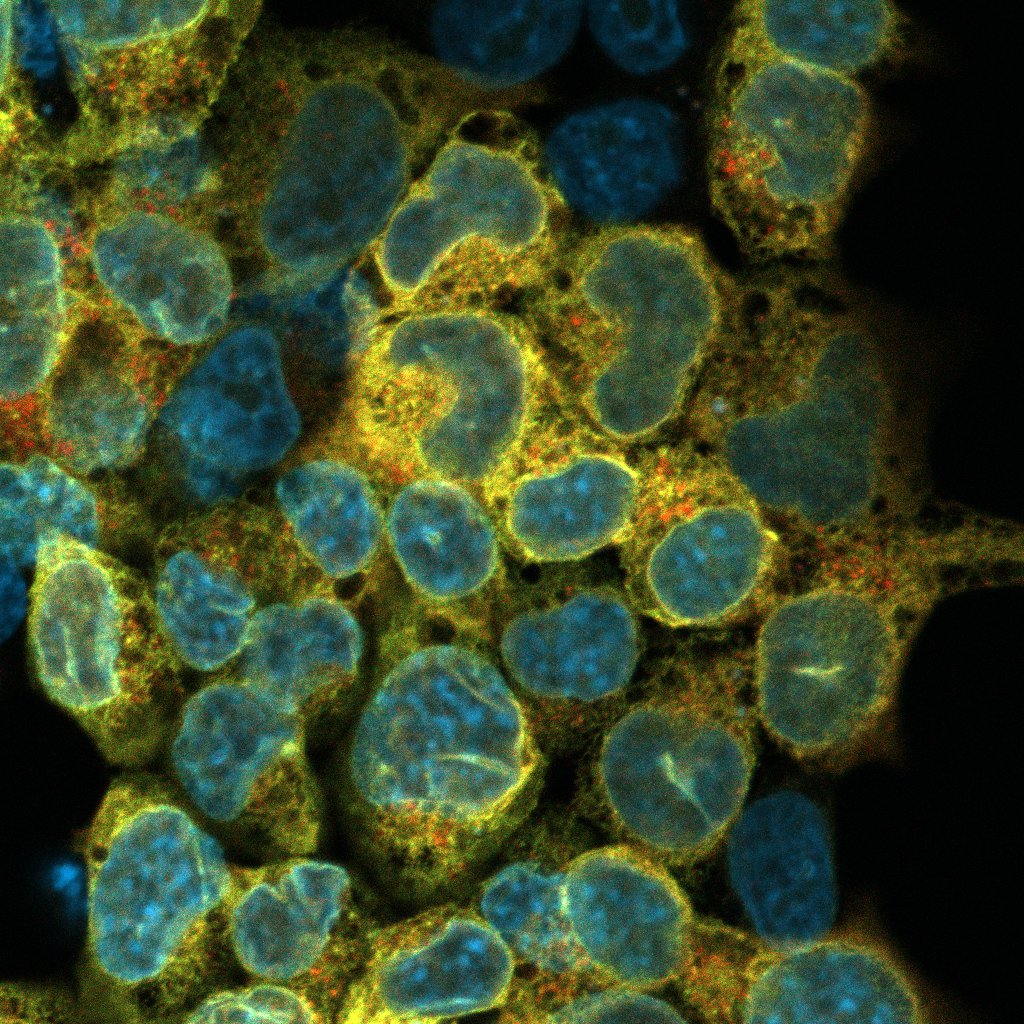

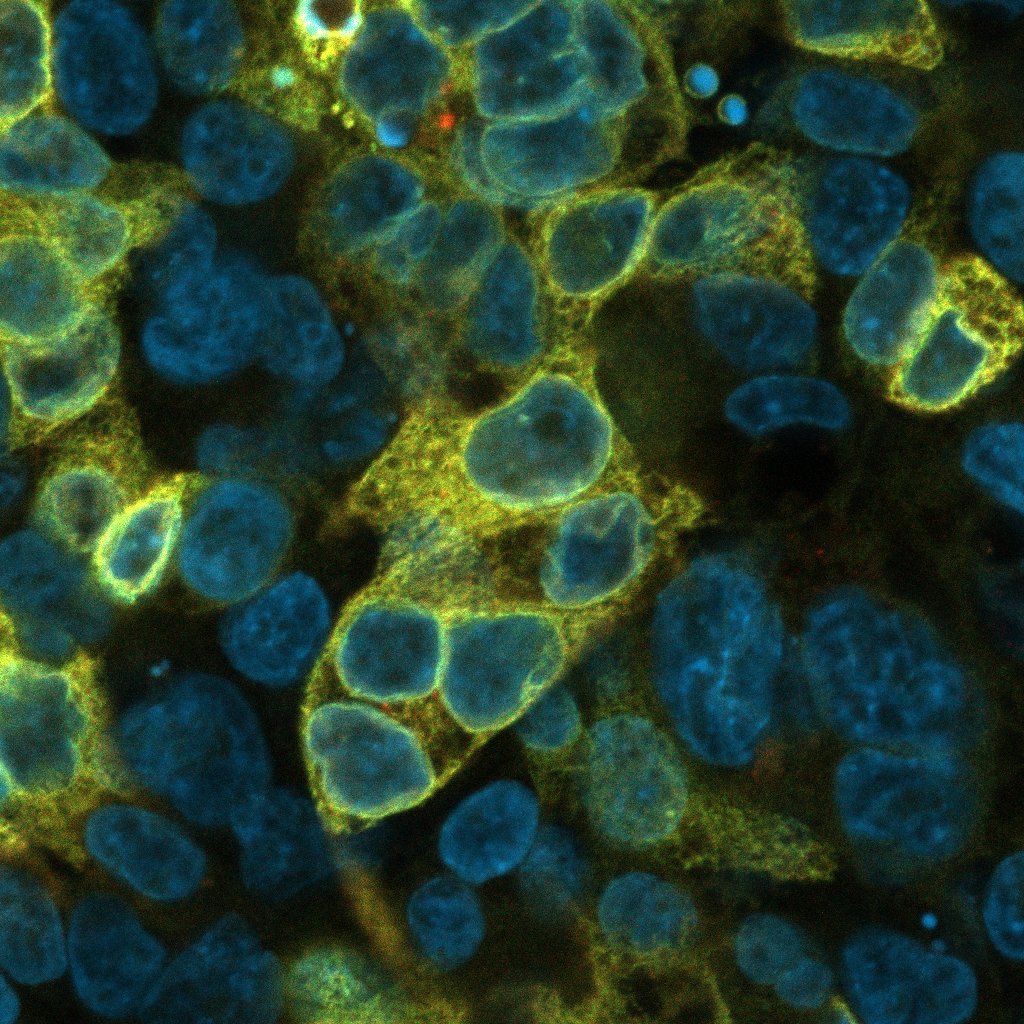


**untreated**

**starvation**

**starvation +**

**Baf A1**

**C**

**GFP**

**mCherry**

**merge**

**Blow-up 5X**

**Figure S6.** Characterization of the HCT116 reticulophagy reporter cell line. (**A**) HCT116 cells were stably transfected with a reticulophagy reporter expressing in-frame the ER-membrane protein SERP1, GFP, and ChFP under the control of a tetracycline-regulated promoter (HCT116-EATR). Immunoblots probed with an anti-GFP antibody confirmed the expression of the fusion protein in cells treated with 2 μg/ml Dox for 24 h. The slower migrating band corresponds in size to the full-length protein. Additional bands are likely to correspond to degradation products whose presence does not interfere with the interpretation of the data. (**B**) Visualization of reticulophagy. Induced HCT116-EATR cells were starved overnight in EBSS medium. Red dots corresponding to ER-loaded autolysosomes were visualized by confocal microscopy. (**C)** Inhibition of lysosome acidification by treatment with Baf A1 resulted in the accumulation of yellow ER-loaded autolysosome in starved HCT116-EATR cells. Scale bar: 10 μm

**BMRF1**

**50**


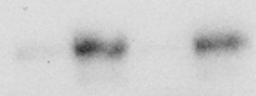

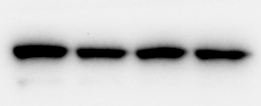


**GAPDH**

**37**

**Dox**

**-**

**-**

**+**

**+**

**LCL-WT**

**LCL-cm**

**A**

**LCL-WT**

**LCL-cm**

**B**


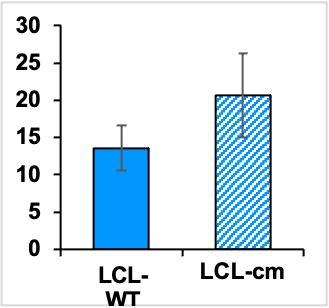


***BPLF1 mRNA***

**(fold induction)**

**C**

**Figure S7.** Induction of the productive virus cycle in the LCL-WT and LCL-CM cell lines. Expression of the BZLF1 transactivator was induced by treatment with Dox for 72 h, and the efficiency of productive virus cycle induction was monitored by probing western blots with the BZLF1 transcriptional target BMRF1. (**A**) Representative western blot illustrating the comparable expression levels of BMRF1. (**B**) Quantification of for independent induction experiments. Relative intensity was calculated as the ratio between the intensity of the BMRF1 band in LCL-WT versus LCL-CM after normalization of the GAPDH loading control. (**C**) qPCR quantification of *BPLF1* mRNA in four independent experiments. Fold increase was calculated relative to the uninduced controls.
